# Intestinal Pathogenic *Escherichia coli*: Insights for Vaccine Development

**DOI:** 10.3389/fmicb.2018.00440

**Published:** 2018-03-20

**Authors:** Maricarmen Rojas-Lopez, Ricardo Monteiro, Mariagrazia Pizza, Mickaël Desvaux, Roberto Rosini

**Affiliations:** ^1^GSK, Siena, Italy; ^2^Institut National de la Recherche Agronomique, Université Clermont Auvergne, UMR454 MEDiS, Clermont-Ferrand, France

**Keywords:** vaccines, intestinal pathogenic *E. coli* (InPEC), Enterohemorrhagic *E. coli* (EHEC), Enterotoxigenic *E. coli*, Enteropathogenic *E. coli*

## Abstract

Diarrheal diseases are one of the major causes of mortality among children under five years old and intestinal pathogenic *Escherichia coli* (InPEC) plays a role as one of the large causative groups of these infections worldwide. InPECs contribute significantly to the burden of intestinal diseases, which are a critical issue in low- and middle-income countries (Asia, Africa and Latin America). Intestinal pathotypes such as enteropathogenic *E. coli* (EPEC) and enterotoxigenic *E. coli* (ETEC) are mainly endemic in developing countries, while ETEC strains are the major cause of diarrhea in travelers to these countries. On the other hand, enterohemorrhagic *E. coli* (EHEC) are the cause of large outbreaks around the world, mainly affecting developed countries and responsible for not only diarrheal disease but also severe clinical complications like hemorrhagic colitis and hemolytic uremic syndrome (HUS). Overall, the emergence of antibiotic resistant strains, the annual cost increase in the health care system, the high incidence of traveler diarrhea and the increased number of HUS episodes have raised the need for effective preventive treatments. Although the use of antibiotics is still important in treating such infections, non-antibiotic strategies are either a crucial option to limit the increase in antibiotic resistant strains or absolutely necessary for diseases such as those caused by EHEC infections, for which antibiotic therapies are not recommended. Among non-antibiotic therapies, vaccine development is a strategy of choice but, to date, there is no effective licensed vaccine against InPEC infections. For several years, there has been a sustained effort to identify efficacious vaccine candidates able to reduce the burden of diarrheal disease. The aim of this review is to summarize recent milestones and insights in vaccine development against InPECs.

## Introduction

*Escherichia coli* is a Gram-negative bacterium commonly found as a commensal in the human microbiota. However, the plasticity of its genome has led to the evolution of this organism into pathogenic strains able to cause diseases and syndromes of public health importance in humans and animals. Pathogenic *E. coli* are mainly divided into two groups depending on the disease location: extraintestinal pathogenic *E. coli* (ExPEC) and intestinal pathogenic *E. coli* (InPEC). While ExPEC strains are associated mainly with neonatal meningitis (NMEC) and urinary tract infections (UPEC) in adults, InPEC strains, related to diarrheal disease, are subdivided into at least 6 well-known pathotypes: enteropathogenic *E. coli* (EPEC), enterohemorrhagic *E. coli* (EHEC), enteroinvasive *E. coli* (EIEC), enteroaggregative *E. coli* (EAEC), diffusely adherent *E. coli* (DAEC) and enterotoxigenic *E. coli* (ETEC). These *E. coli* enteropathotypes are classified according to their virulence factors, mechanisms of infection, interaction with the enterocyte, tissue tropism, symptoms and syndromes (Kaper et al., 2004; Croxen and Finlay, 2010). Most recently, adherent-invasive *E. coli* (AIEC) have also been described as a disease-associated *E. coli* related to Crohn's disease, but they cannot strictly be considered as diarrheagenic *E. coli* and so are classified as a new InPEC pathotype.

Nowadays, the burden of diarrheal diseases caused by several etiological agents is one of the major public health problems around the world in the absence of preventive strategies such as vaccines to block infections. Besides InPEC, these diarrheagenic agents include *Cryptosporidium, Campylobacter, Shigella* and *Salmonella*, which are responsible for the vast majority of diarrheal diseases worldwide (MacLennan and Saul, 2014). In order to standardize the real estimation of the burden of diarrheal disease, the etiology and the real number of the most affected populations, mathematical approaches have been developed. The Global Burden of Disease (Institute for Health Metrics and Evaluation, 2013) and Child Health Epidemiology Reference Group (CHERG by World Health Organization) indexes have been implemented (Kovacs et al., 2015) to calculate numbers in the burden of diarrheal disease in children under 5 years old (U5). The main goal of these approaches is the estimation of cause-specific disease and morbidity-mortality, but calculations yield different numbers because of the inclusion of diverse data. In addition, the Global Enteric Multicenter Study (GEMS), a case-control study still ongoing in Africa and Asia, is designed to identify the etiology and population-based burden of pediatric diarrheal disease (Kotloff et al., 2013). Overall, these approaches will allow us to refine regulations and therapies, control strategies and estimate the real numbers of infectious diseases, depending on the specific target (Pires et al., 2015).

InPECs contribute significantly to the burden of diarrheal diseases, which represent a critical issue in low- and middle-income countries (in Asia, Africa and Latin America). For instance, EPEC and ETEC are mainly endemic in developing countries and the latter is the main cause of diarrhea in travelers to these countries (Kaper et al., 2004; Torres, 2017a), whereas EHEC strains are responsible for large outbreaks around the world. This pathotype, mainly affecting developed countries, not only causes diarrheal disease, but is also responsible for clinical complications like hemorrhagic colitis and hemolytic uremic syndrome (HUS), which is an increasing problem in Latin American countries like Argentina (Kaper et al., 2004; Pianciola et al., 2016; Torres, 2017a). Similarly, EAEC has also occasionally been involved in diarrheal diseases in developing and industrialized countries (Foster et al., 2015) and in 2011 a EHEC-EAEC hybrid caused a large outbreak in Europe with 3,816 reported cases, leading to 845 HUS cases and 54 deaths (Brzuszkiewicz et al., 2011; Frank et al., 2011).

Overall, (i) an increase in the burden of the disease, because there is a high incidence of traveler diarrhea (ETEC, EAEC), (ii) endemic ETEC and EPEC cases in developing countries, (iii) infections with EHEC and an increasing number of HUS episodes, (iv) the annual cost for the healthcare system, (v) the emergence of antibiotic resistant strains arise the need for effective preventive treatment to reduce the burden of diarrheal disease. Although the use of antibiotics is still key to the treatment of such infections, non-antibiotic strategies are either a crucial option to limit the increase in antibiotic resistant strains (Torres, 2017a) or the only option for diseases where antibiotic therapies are not recommended, e.g., EHEC infection (Goldwater and Bettelheim, 2012; Rivas et al., 2016). Recently, preventive methods to reduce the risk of ETEC-induced diarrhea have been proposed. A hyperimmune bovine colostrum (HBC) produced by immunization of cows during gestation has proven clinically effective in preventing ETEC diarrhea (Sears et al., 2017). Among non-antibiotic therapies, vaccine development is a strategy of choice but, to date, there is no universal or specific licensed vaccine against InPEC.

For years, vaccine development has included various platforms and approaches, such as (i) pathogens attenuated by exposure to different environmental conditions (heat or oxygen) or by multiple passages in culture media (*in vitro*), a method considered as the most ancient and empirical form of vaccine production; (ii) detoxified toxin forms, like the detoxified version of the diphtheria and tetanus toxins; (iii) the use of protein-based vaccines, as hemagglutinin from influenza virus or the vaccine for *Bordetella pertussis*; (iv) genetically engineered vaccines, which have been the most used alternative to vaccine development, in which antigens can be produced in different vectors that reduce toxicity or collateral immunoreactions (Mora et al., 2003; Plotkin, 2014).

However, there are several infectious diseases for which these traditional approaches have failed and for which vaccines have not yet been developed. With the advent of whole-genome sequencing and advances in bioinformatics, the vaccinology field has changed. Approaches like reverse vaccinology, based on the scanning of the annotated complete pathogen genome by bioinformatic prediction of the most likely vaccine candidates, have enabled identification of promising antigens and the development of a safe broadly protective vaccine against the *Neisseria meningitidis* serogroup B pathogen (Pizza et al., 2000; Giuliani et al., 2006; Feavers and Maiden, 2017). For other pathogens such as *Streptococcus ssp*., and for extraintestinal pathogenic *E. coli* (ExPEC), a number of promising antigens have also been identified (De Gregorio and Rappuoli, 2012; Sjoling et al., 2015). In particular, for ExPEC the genome sequence analysis of a neonatal meningitis isolate (NMEC) enabled the identification of 230 potential antigens. The most protective antigens revealed by that analysis were a broadly conserved adhesin (FdeC) and a conserved secreted zinc metallopeptidase (SslE), which conferred cross-protection in three different murine models, including intestinal, ascending urinary tract infection and sepsis models (Moriel et al., 2010; Nesta et al., 2012, 2014). Using the reverse vaccinology approach on 1,700 draft and complete genomes, a conserved and protective vaccine candidate was recently identified. This potential antigen, a seven-bladed beta-propeller protein (YncE), was found to be present in and widely expressed by different *E. coli* strains, including ETEC, EHEC, EAEC, and UPEC pathotypes (Moriel et al., 2016).

**Figure 1:**
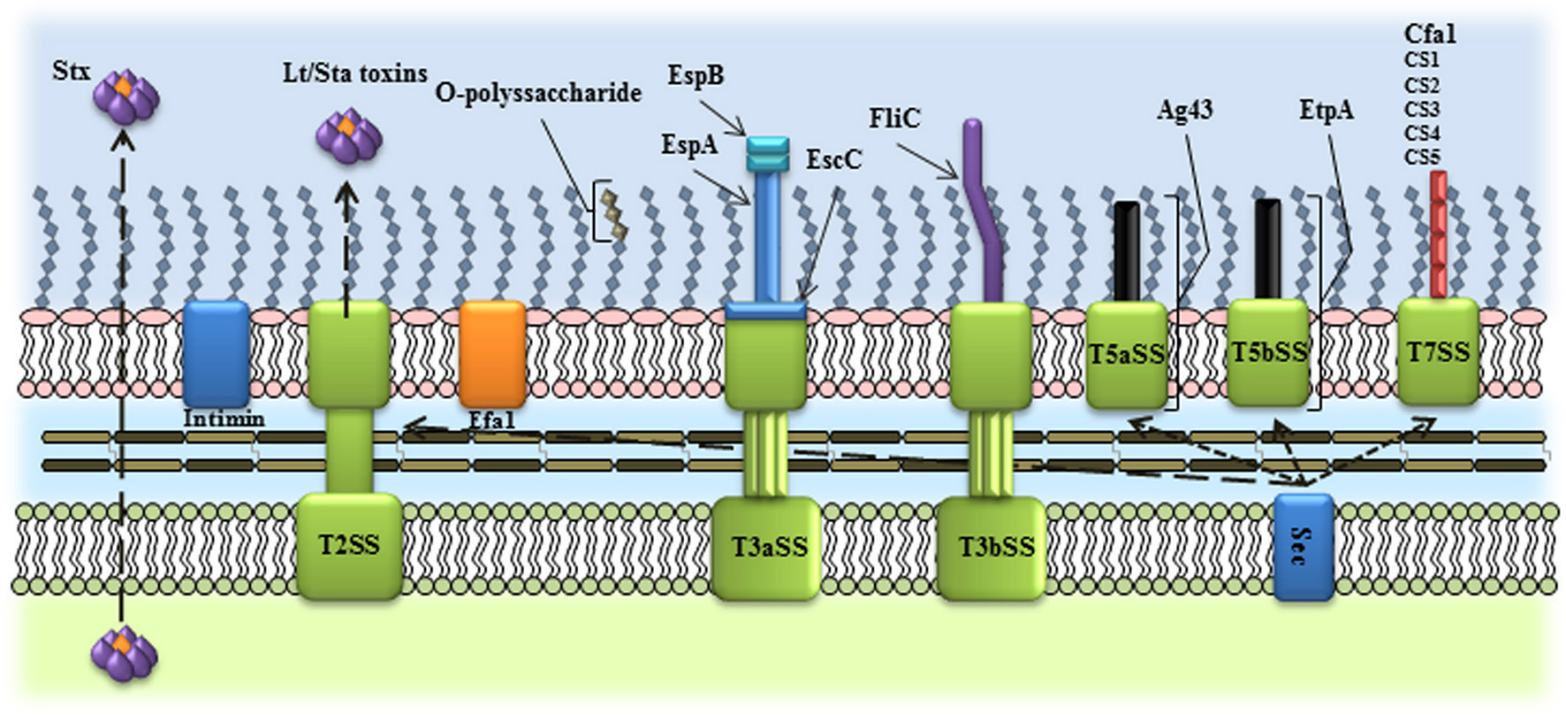
Schematic representation of the virulence factors used as vaccine candidates in InPEC. Several InPEC virulence factors have been employed as vaccine candidates, including outer membrane proteins, toxins, O-polyssaccharides, exported and secreted proteins. T2SS, type II secretion system; T3aSS, type III, subtype a, secretion system; T3bSS, type III, subtype b, secretion system; T5aSS, type V, subtype a, secretion system; T5bSS, type V, subtype b, secretion system; T7SS, type VIII secretion system.

Significant efforts to identify effective vaccines against InPEC have been made for several years by different research groups worldwide. To date, the major pathotypes considered for vaccine development are EHEC and ETEC, because of their major impact on the public health burden (Table 1), even though EPEC is a major problem in developing countries, little progress has been made. The aim of this review is to summarize the advances made in the development of vaccines against these different InPEC pathotypes and the perspectives they offer (Figure 1).

**Table 1:** Summary of the most promising vaccine candidates against EHEC and ETEC.

**Type**	**Key component**	**Efficacy outcome**	**References**
**EHEC**			
Shiga toxin-based vaccines	Antibodies cαStx1B and cαStx2A	Good tolerance and safety in a human trial single dose study	Bitzan et al., 2009
	Two copies of anti-Stx2B VHH and one anti-serum albumin VHH	Decreased toxicity in Stx2 lethal mouse model	Mejias et al., 2016
Attenuated bacteria-based vaccines	EHEC O157:H7 86-24 strain Δ*ler*Δ*stx2* expressing Stx1A Stx2A detoxified	Reduced EHEC O157:H7 wild-type strain colonization	Liu et al., 2009
	Attenuated EPEC O126:H6	Reduced mortality in mouse model and cross-reaction of produced EspB and intimin EPEC antibodies with EspB and intimin from EHEC	Calderon Toledo et al., 2011
	Attenuated *Salmonella typhimurium* expressing recombinant EspA, intimin and Stx2B	Higher titer of specific antibodies, and specific lymphocyte proliferation	Oliveira et al., 2012
	Attenuated *Salmonella typhimurium* χ3987 (Δ*cya*,Δ*crp*,Δ*asd* and H683 Δ*aro*Δ*asd*) expressing γ-intimin variant	Induces systemic and humoral immunity by increased titers of IgG in serum and IgA in feces. Reduced EHEC O157:H7 shedding post-challenge	Oliveira et al., 2012
	Recombinant bacillus Calmette-Guérin expressing Stx2B (rBCG-Stx2B)	Increased levels of Stx2 IgG in mice. Higher survival rate (63%) in immunized mice after EHEC challenge	Fujii et al., 2012
Bacterial ghost-based vaccines	Bacterial ghosts of O157:H7 unable to cause infection	Antitoxicity effect on Vero cell culture. Stops shedding of EHEC O157:H7 wild-type strain and survival rate of 93% in orally immunized mice and 100% in rectally immunized mice	Mayr et al., 2012
	Bacterial ghosts of O157:H7 exposing Stx chimeric protein (Stx2Am-Stx1B)	High specific IgG and IgA antibody titers to Stx1A and Stx2B. Survival rate of 52% in immunized mice	Cai et al., 2015
Protein-based vaccines	EspA-Stx1A fusion protein	High titers of specific IgG antibodies to EspA-Stx1A and 95% survival in mice after a challenge with crude toxin Stx2	Cheng et al., 2009
	Stx1B-Stx2-truncated intimin fusion protein	100% survival rate in orally immunized mice challenged with EHEC O157:H7 88321 and anti-toxin and anti-adhesion effect	Gansheroff et al., 1999
	Stx2Am-Stx1B, SAmB fusion protein	93% survival rate of orally immunized mice challenged with EHEC O157:H7 88321	Gao et al., 2011
Peptide-based vaccines	C terminal region of intimin	Reduces bacterial attachment to Hep-2 cells and confers protection on immunized mice	Wan et al., 2011
	Peptide KT-12 (KASITEIKADKT) conjugated with KLH	High concentrations of IgG in subcutaneously immunized mice and high titers of IgA in intranasally immunized mice	Zhang et al., 2011
Plant-based vaccines	*Nicotiana tabacum* (tobacco) NT-1 cell line expression inactivated Stx1A	High specific IgA anti Stx2 in fecal samples from orally immunized mice, conferring high protection against STEC strain B2F1 (75% survival rate)	Wen et al., 2006
	Chimeric gene *espA*-*eae*-*tir*	Reduction of EHEC O157:H7 shedding, colonization and histological damage in subcutaneously or orally immunized mice	Amani et al., 2009
DNA-based vaccines	Stx2AΔAB DNA vaccine	Protection of immunized mice challenged with native Stx2 and toxin neutralization in Vero cell culture.	Bentancor et al., 2009
	C-terminal domain of *EscC*	Reduced bacterial counts in feces, colon and cecum and increased IgGs in sera and IgA in feces from intranasally immunized mice	Garcia-Angulo et al., 2014; Tapia et al., 2016
	pVAX-efa1 (*efa-1′*)	High levels of specific mucosal IgA and reduction of EHEC colonization	Riquelme-Neira et al., 2015
Polysaccharide-based vaccines	O-specific polysaccharide of EHEC O157:H7 conjugated with recombinant exotoxin A of *P. aeruginosa* (O157-rEPA)	High levels of IgG against LPS in vaccinated children with non-collateral reactions to the vaccine	Konadu et al., 1994; Ahmed et al., 2006
Adjuvant enhanced vaccines	EspB and/or C-terminal of γ-intimin proteins + MALP-2 adjuvant	Higher bronchoalveolar titers of IgA	Cataldi et al., 2008
	Chimeric protein Tir-Stx1B-Stx2B + Zot adjuvant	Protection against EHEC. High IgA and IgG response and reduction of bacterial shedding in feces post-EHEC wild-type challenge in subcutaneously immunized mice	Zhang et al., 2011
**ETEC**			
Toxin-based vaccines	LT toxin using skin patch	Increased anti-LT IgG and IgA in 97–100% of human volunteers	McKenzie et al., 2007
	STaP13F-LTR192G toxoid fusion protein	Development of IgG specific antibodies for LT and STa proteins in serum and feces and only IgA in feces in immunized mice	Liu et al., 2011
Autotransporter-based vaccines	Recombinant Ag43 and pAT autotransporters	Increased fecal IgA and relative protection against intestinal colonization in immunized mice	Harris et al., 2011
Adhesin-based vaccines	Recombinant ETEC two-partner secretion protein A (EtpA)	Inhibition of ETEC colonization in immunized mice	Roy et al., 2009
	CS21/LngA formulated with cholera toxin	Increased specific IgG antibodies in serum and IgA antibodies in fecal and intestinal lavages and stopped bacteria shedding in immunized mice	Zhang et al., 2016
Attenuated bacteria-based vaccines	ETEC E1392/75-2A Δ*aroC*Δ*ompR* and ETEC E1392/75-2A Δ*aroC*Δ*ompRΔompC*	IgA and IgG response and specific antibodies against CS1 and CS3	Turner et al., 2001; McKenzie et al., 2006
	Etvax (attenuated bacteria expressing CS6 in K12 and CFA/I, CS3, CS5 in ETEC O78 toxin-negative) + LCTBA hybrid protein + double mutant LT	High titers of fecal, jejunal and serum IgA and IgG in orally immunized humans	Norton et al., 2011
	*Vibrio cholerae* strains expressing CFA/I operon (cfaA-B-C-E)	Increased IgA and IgG titers in serum of immunized mice.	Tobias et al., 2008
	ETEC strains expressing CFA/I (ACAM2010 str.), CS2 and CS3 (ACAM2007 str.) and CS1, CS2, and CS3 (ACAM2017 str.)	Increased IgA against CFA level in orally immunized human volunteers	Daley et al., 2007
	Non-toxigenic *E. coli* expressing CS2, CS4, CS5, or CS6 and CFA/I	Induced IgG+IgM antibodies against CS6 in sera and IgA in fecal samples of immunized mice	Tobias et al., 2010
	ACE527 ETEC complex (ACAM2022 (O141:H5, expressing CS5 and CS6), ACAM2025 (O39:H12, expressing CFA/I) and ACAM2027 (O71:H-, expressing CS2, CS3, and CS1)	Helps shorten the duration of diarrhea and reduce shedding of the wild-type strain, conferring a protection level ranging from 33% to 98% in human clinical trials	Harro et al., 2011a,b
OMV-based vaccines	ETEC OMVs Δ*msbB*Δ*eltA*	Detoxified OMV yielded higher titers of IgG1, IgM, and IgA and reduced wild-type colonization in immunized mice	Leitner et al., 2015
	*Vibrio cholerae* OMV Δ*msbB*Δ*ctxAB*Δ*flaA* expressing ETEC FliC and CFA/I	OMV yielded higher titers of IgG1, IgM, and IgA and reduced wild-type colonization in immunized mice	Leitner et al., 2015

## Development of vaccines against EHEC

As for each of the characterized InPEC, EHEC is primarily defined by an array of evidence based on clinical manifestations, i.e., clinical symptoms, and histological and molecular features (Nataro and Kaper, 1998; Kaper et al., 2004). One of its primary features is the presence of Shiga-toxin (Stx) genes, so EHEC belongs to the genotypic group of Shiga-toxin encoding *E. coli* (STEC). While there are over 380 distinct serotypes of STEC (Karmali et al., 2003, 2010), the EHEC serotype O157:H7, as well as the big six non-O157 serotypes O26:H11, O45:H2, O103:H2, O111:H8, O121:H19 and O145:H28, are the most frequently associated with human disease and large outbreaks around the world (Karmali et al., 2003, 2010). Based on their incidence, involvement in outbreaks and association with severe disease, STEC can be classified into 4 classes, namely (i) class A corresponding to the O157 serogroup with a high virulence, (ii) class B corresponding to the big six non-O157 serotypes mentioned above with a moderate virulence, (iii) class C corresponding to serotypes mainly involved in some sporadic cases with a moderate virulence, and (iv) class D corresponding to serotypes never reported in human infection and thus avirulent (Karmali et al., 2003). In this regard, EHEC clearly is defined as a subset of clinically isolated pathotypes of InPECs, but STEC isolates do not necessarily refer to a specific intestinal pathogenic *E. coli* pathotype (Nataro and Kaper, 1998). There is currently no simple answer to the question of whether a STEC strain isolated from the environment or food, for instance, is truly an EHEC, and whether it is virulent (and to what degree) and a risk for human health (Messens et al., 2015). In the United States since 2014, the number of cases per 100,000 population was 690 for EHEC O157 and 445 for EHEC non-O157, while 16% of outbreaks were accounted for by EHEC O157 and 7% by EHEC non-O157 (Crim et al., 2015). The incidence of EHEC non-O157:H7 has increased and it has become a human pathogen as important as EHEC O157:H7 (Croxen et al., 2013). More recently, infection with EHEC strains is rising in Latin American countries and has become an endemic phenomenon, as in Argentina (Mejias et al., 2016).

As asymptomatic carriers, cattle are the natural reservoir of EHEC and, as a zoonosis, infection can occur from direct (meat, milk) or indirect (vegetables, water) contamination of food products by animal feces (Rivas et al., 2016). The adhesion and persistence of the bacteria is an important key feature in bacterial pathogenesis. EHEC O157:H7 mainly colonizes the human colon and carries a pathogenic island (PAI) known as the Locus of Enterocyte Effacement (LEE) (like EPEC), which encodes a Type III, subtype a, secretion system (T3aSS). T3aSS would give the capability to produce attaching and effacing (A/E) lesions in humans, but this phenomenon is mainly observed in *in vitro* assays more than *in vivo*. However, the PAI LEE is still a determinant factor for colonization and persistence in other reservoirs (e.g., cattle) and its presence is more associated with pathogenic strains (Nataro and Kaper, 1998; Coombes et al., 2011; Lewis et al., 2015). Interestingly, the EHEC non-O157:H7 strains do not necessarily carry the LEE PAI. Other colonization factors involved in the attachment, persistence and tissue tropism from EHEC O157 and non-O157 have been well described, including Lpf, Ecp, Hcp, F9, curli, Type 1 fimbriae, Autotransporters (EhaA, EhaB), EspP, Saa, Cah (McWilliams and Torres, 2014; Monteiro et al., 2016).

Besides the adhesion factors, toxins play a key role in EHEC pathogenesis and, as mentioned above, Stx is one of the important virulence factors. This family of AB toxins is divided into Stx1 and Stx2 (with allelic variants); although both toxins or just one can be produced by the strain, Stx2 is considered one of the more potent toxins, and thus more related to O157 infections. It has been shown that the use of antibiotics in the treatment of EHEC infections could lead to cellular damage by increasing the production of this toxin, activating the SOS system and by disrupting the bacterial membrane, causing release of the toxin (Kimmitt et al., 2000). The resulting secretion of the toxin into the blood stream could lead to worsening of the disease (Pacheco and Sperandio, 2012). The Stx toxin is one of the factors involved in development of HUS, which is characterized by the triad of microangiopathic hemolytic anemia, thrombocytopenia, and acute renal failure, with a 10% additional chance of HUS development in children less than 10 years of age. Besides triggering HUS and later renal failure, Stx toxin is also responsible for strokes (Tarr et al., 2005).

Due to the involvement of Stx toxin during the course of the infection and to restrictions in treatment, different preventive strategies, such as vaccines, have been implemented and are exemplified in the following sections.

### Development of Stx-based treatments

Neutralizing the effect of the toxin has been one of the strategies to reduce its effect. Antibodies cαStx1 and cαStx2 have been directly engineered against the B subunit of Stx1 and A subunit of Stx2, respectively (Bitzan et al., 2009). Tolerability and the pharmacokinetic profile have been investigated using chimeric anti-Stx1 and anti-Stx2 antibodies. This combination comprises the variable regions of the murine Stx1-neutralizing or Stx2-neutralizing monoclonal antibodies (mAbs) 13C4 or 11E10, respectively, fused to human kappa light chain constant-domain sequence and human immunoglobulin G1 (IgG1) heavy chain constant-domain sequence. These antibodies were well tolerated and safe as antitoxins in healthy human volunteers in a single-dose clinical trial study. Although it has been shown that these mAbs neutralize the effect of Stx toxins in mice, it remains to be established whether they are able to avoid the development of HUS after diarrhea caused by EHEC (Bitzan et al., 2009).

More recently, camelid single antibodies against Stx2 have been tested for their protective characteristics against Stx2 (Mejias et al., 2016). The anti-single chain antibodies (VHH) were obtained with two copies of anti-Stx2B VHH and one anti-serum albumin VHH. This trivalent molecule, administered to mice, decreased toxicity in a Stx2 lethal mouse model. Because of the antitoxin effect of VHH, it is proposed as an alternative for treatment of HUS sequelae (Mejias et al., 2016).

### Attenuated bacteria and bacterial ghost platform system

Gene regulators (global or specific) are important key players for the expression or silencing of virulence factors in pathogens. In fact, deleting those that promote the expression of specific virulence factors could be useful strategy to attenuate a pathogen. This approach has been applied to EHEC by deleting the LEE-encoded regulator (Ler). Ler is an important specific regulator that positively controls the expression of LEE genes involved in the A/E phenotype, as well as genes outside the PAI, including adhesins and genes in the plasmid pO157. An EHEC O157:H7 86-24 strain deleted in *ler* and *stx*2 genes (ler/Stx2) but carrying a plasmid that expresses a detoxified version of the Stx1A subunit and Stx2A subunit was used as an attenuated vaccine candidate. These bacteria with reduced toxicity and safe for animal administration were used to immunize mice via IP. Animals were challenged using EHEC O157:H7 wild-type strain and after 6 days the wild-type strain was not detected in feces, indicating a capability of the attenuated strain to reduce wild-type colonization. Also, there was a 70% survival rate in passively immunized suckling mouse offspring born to dams previously immunized with this vaccine, after a challenge with wild-type EHEC bacteria (Liu et al., 2009).

EPEC is the closest pathotype to EHEC: both express a T3aSS element and cognate secreted proteins such as EPEC-secreted proteins (Esps) and intimin (important for the A/E phenotype). It is worth noting that *E. coli*-secreted protein B (EspB) is responsible together with *E. coli*-secreted protein D (EspD) for forming a pore structure in eukaryotic cells, which allows the translocation of effector proteins through the needle formed by homodimers of *E. coli*-secreted protein A (EspA) (Garmendia et al., 2005). Using an EPEC as live attenuated vaccine to assess the cross-protection among the two pathotypes, only partial protection against EHEC was shown. In fact, intragastric immunization with a clinical isolate EPEC O126:H6 and challenge using the EHEC O157:H7 wild-type strain yielded sick mice, but with no dead mice reported. Interestingly, it was also shown that the EspB and intimin antibodies produced after the EPEC vaccination were cross-reactive against EspB (translocated protein and effector that prevents phagocytosis) and intimin from EHEC (Calderon Toledo et al., 2011).

Using a different delivery system such as attenuated *Salmonella* strain, recombinant EspA (300-amino-acid carboxyl-terminal), intimin and the B subunit of Stx2 proteins were expressed. Mice either orally immunized only or orally immunized and subcutaneously boosted were able to raise specific IgG and IgA antibodies against these three antigens at similar levels. However, specific anti-intimin antibody levels were higher in mice orally immunized and subcutaneously boosted than in mice that were orally immunized only. Yet, IgG antibodies specific for Stx increased a week after the booster vaccination; IgA specific for Stx2B antibodies were only detected in feces and increased even more when boosted (Gu et al., 2011).

Similarly, an attenuated strain of *Salmonella* enterica serovar Typhimurium χ3987 (Δ*cya*, Δ*crp*, Δ*asd* and H683 Δ*aro* Δ*asd*) expressing heterologous proteins for EHEC such as γ-intimin variant, encoded by the *eae* gene, was used as a vaccine. The vaccination using these attenuated bacteria increased titers of IgG in serum and IgA in feces, indicating an immune response from systemic and humoral immunity. The attenuated bacteria expressing intimin were still detectable in Peyer's patches and spleen, while in feces CFUs decreased in number from day 2 to 10 and subsequently were constant. Animals immunized with the attenuated *Salmonella* had reduced EHEC O157:H7 shedding post-challenge and increased production of IgA and IgG (Oliveira et al., 2012).

A delivery antigen mucosal system used for EHEC vaccine development was based on Bacillus Calmette-Guérin (BCG) (a live attenuated strain of *Mycobacterium bovis*), because of its mucosal humoral immunoreactivity. A recombinant rBCG-Stx2B, expressing the Stx2 subunit B, was generated and then used to immunize mice. This recombinant bacillus was able to increase the levels of Stx2 IgG in serum that were directly proportional to the number of CFUs. The protection was confirmed when after challenge using a wild-type EHEC strain there was a higher survival rate (63%) in immunized mice with higher CFU concentrations than in immunized mice with fewer CFUs of rBCG-Stx2 or null rBCG (Fujii et al., 2012).

Among attenuated bacteria strategies, a delivery system based on a bacterial ghost (BG) platform for vaccine development against EHEC has been proposed. Bacterial ghosts of *E. coli* O157:H7 have been generated by the controlled expression of the X174 lysis gene. This gene produces empty bacterial cell envelopes with the composition of the cell envelope of a living cell, such as LPS, lipids, peptidoglycans (acting as adjuvants), but which lack the capability to produce infection. These bacteria had an antitoxicity effect on cultures of Vero cells and were safe for administration in mice (Mayr et al., 2005; Cai et al., 2010). Animals orally immunized twice (days 0 and 28) with BG and then challenged (at day 55) stopped the shedding of bacteria after day 3 post-EHEC and showed a survival rate of 93% (Cai et al., 2010). In addition, rectal immunization showed a 100% survival rate and reduced bacterial shedding for 3–5 days, thereby protecting mice against EHEC O157:H7 after challenge (Mayr et al., 2012).

However, mice that were orally or intragastrically immunized still showed disease symptoms from days 2 to 7 post-challenge, including anorexia, slowing of activity, no stimulus reaction, and convulsions before death (Cai et al., 2010). Data also showed that surviving mice recovered after 7 days and dead mice developed glomerulus necrosis and enterocyte effacement. Interestingly, specific IgA and IgG antibodies titers were raised in serum and colon from mice immunized twice orally with BG (Cai et al., 2010). In fact, the vaccination of mice with BG is, *per se*, able to trigger the immune response, as observed in Th1/Th2 cell proliferation and higher interferon gamma (INF-γ) levels in spleen cells, leading to increased titers of IgG and IgA, in both serum and colon samples (Mayr et al., 2005, 2012; Cai et al., 2010).

More recently, a BG (rSOBG) expressing an Stx chimeric protein composed of the Stx2A and Stx1B subunits (Stx2Am-Stx1B) has been engineered. This rSOBG showed specific IgG and IgA antibody titers to StxA1 and StxB2, and the rate of survival was higher (52%) than with native bacterial ghost-OBGs (12%) when mice were challenged intragastrically with high doses of viable *E. coli* O157:H7. Also, there was no tissue damage in the liver, kidney or intestine of rSOBG-immunized mice (Cai et al., 2015).

### Protein-based vaccines

Among several strategies applied for the development of vaccines against EHEC, chimeric protein constructions have proved to be an attractive approach in recent years. In this regard, a fusion protein of the A1 subunit of Stx2 toxin and the N-terminus of EspA has been tested for its immunoreactivity. Mice subcutaneously immunized with EspA-Stx2A1 fusion protein showed high titers of IgG antibodies specific to EspA-Stx2A1. This humoral immune response resulted in >95% of survival after a challenge with crude toxin Stx2. Although *in vitro* assays on HeLa cells showed that the anti-EspA-Stx2A1 serum was able to neutralize the action for Stx2, it did not prevent the adherence of bacteria to HeLa cells (Cheng et al., 2009).

Another protein fusion constructed with the subunit B from two Shiga-toxins Stx1/Stx2 and a truncated intimin protein (SSI) increased specific IgG antibody titers in mice. The immunized mice orally challenged with EHEC O157:H7 88321 showed a 100% survival rate. However, protection using chimeric vaccines depends on the number of immunizations and the bacterial challenge dose (Cai et al., 2011). Immunization with this SSI chimeric protein avoided pathological damage to colon and kidney tissues, generating antibodies with anti-toxin and anti-adhesion effects, which were absent with the vaccines using single proteins (Gansheroff et al., 1999; Gao et al., 2009). Even though the toxins could have contributed to an adjuvant and neutralization effect, the fusion protein was not able to avoid wild-type adhesion in *in vitro* assays, as with other subunit vaccines (Gansheroff et al., 1999). A vaccine composed of an Stx1B subunit and an enzyme-inactive Stx2A subunit (Stx2Am-Stx1B, SAmB) induced a Th2-mediated humoral immune response and its typical cytokines, IL4 and IL 10, but a low level of INF-γ. Mice immunized with this chimeric protein and challenged with a lysed EHEC 88321 preparation showed 93% survival, and even higher rates of survival were obtained challenging mice with the Stx1, Stx2 or Stx1/Stx2. However, disease manifestations were still evident (Gao et al., 2011).

Specific peptides have been designed for protection against EHEC. An example is the C terminal region of intimin associated with A/E lesions. Antibodies obtained from vaccination with this fragment reduced bacterial attachment to Hep-2 cells cultured *in vitro* and were also associated with protection in mice infected with *E. coli* O157:H7 (Wan et al., 2011). B-cell epitopes of this protein were predicted by structural and antigenicity analysis, and proposed as synthetic vaccine candidates for EHEC (Wan et al., 2011). A promising peptide, KT-12 (KASITEIKADKT) conjugated with KLH, was used to immunize mice by either the subcutaneous (SC) or intranasal (IN) route. Both routes induced high concentrations of IgG, but levels were higher with SC immunization. By contrast, the IgA titer was higher with IN immunization. Although this peptide did not fully protect mice infected with the bacteria, it triggered the immune response (Zhang et al., 2011).

### Plant-based vaccines

In vaccine development, safety is one of the important issues. One approach to reducing the risk of undesired side effects is the use of plant-based vaccines targeting mucosal immunity. The rationale for using plant cells is based on the idea of protecting the antigens from protease degradation in the gastrointestinal tract by means of a plant microencapsulation system, safe oral delivery and at a low production cost (Wen et al., 2006; Amani et al., 2009). An example was provided by a Stx toxoid generated by inactivating the toxin subunit A and expressed in the *Nicotiana tabacum* (tobacco) NT-1 cell line. Mice immunized either orally, by feeding them with these cells expressing the toxoid, or by parenteral immunization and boosted orally, showed elevated specific anti-Stx2 IgA in fecal samples, but levels were higher in the orally immunized mice. Furthermore, sera of immunized mice neutralized Stx2 toxicity in Vero cell cultured, but with a higher neutralization titer using the sera from orally immunized mice (Wen et al., 2006).

Another example of synthetic genes from EHEC was the chimeric gene composed of *espA, eae*, and *tir* antigens (EIT). This gene was codon optimized for expression in plant cells and cloned into a plant-expression vector, using CaMV35S (cauliflower mosaic virus 35S) under the control of FAE promoters for tobacco and canola plants. The EIT protein was used to immunize mice either subcutaneously or orally, and later challenged with *E. coli* O157:H7, resulting in a significant reduction of bacterial shedding. These immunized mice had increased levels of anti-EIT IgG and IgA and reduced bacterial colonization and histological damage (Amani et al., 2009, 2010, 2011).

### DNA vaccines

DNA vaccines have been used to avoid the use of pathogens or bacterial traces that could lead to the development of disease in the vaccine recipient. A DNA vaccine construct encoding the entire StxB2 subunit plus the last 32 amino acid residues of StxA2 was generated and cloned into the pGMS-CSF plasmid encoding the gene for murine granulocyte-macrophage colony-stimulating factor (GMS-CSF). The Stx2AΔAB DNA vaccine expressing the nontoxic Stx2 mutated form increased IgG antibody titers and also conferred protection in immunized mice challenged with native Stx2. In addition, antibodies raised in mice conferred toxin neutralization in Vero cell cultures (Bentancor et al., 2009).

More recently, a selection of prospective DNA vaccine candidates was performed by bioinformatic analysis of EHEC O157:H7: EDL933 and Sakai strain genomes. The vaccine selection included, among others, a putative pilin subunit gene (Z1538), the gene of a T3aSS structural protein (*escC*), the C-terminal side of *escC*, and the gene encoding an outer membrane protein (*iomW*) (Garcia-Angulo et al., 2014; Tapia et al., 2016). Mice were immunized intranasally, and then challenged with the wild-type bacteria. In comparison to the entire *escC* gene, only its C-terminal portion resulted in a greater reduction of bacterial counts in feces, colon and cecum and also in triggering IgG in sera and IgA in feces. The most interesting finding of this study was that the efficacy and immune protection of a vaccine candidate depends on its length and how it is presented to the immune system. In fact, the immune protection against EHEC was improved when only the C-terminal domain of EscC was used (Garcia-Angulo et al., 2014; Tapia et al., 2016).

Another DNA vaccine candidate used was the lymphocyte inhibitory factor A-/EHEC for the adherence-1 gene (*lifA*/*efaA*). This gene encodes a 360 kDa toxin mainly found in non-O157 EHEC strains and associated with LEE strains. It is exposed on the surface of EPEC bacteria and may play a role in colonization and adhesion by mucosal immunity regulation. This gene was originally found as a truncated form in EHEC O157:H7 EDL933 and annotated as *efa-1*′. EHEC carrying the truncated form showed reduced adhesion to human colon cells, showing that Efa-1′ protein still has a role in adhesion. Furthermore, mice vaccinated with this *efa-1*′ showed IgM, IgG and IgA antibody titers. Intranasal immunization using pVAX-efa1 yielded higher levels of antigen-specific mucosal IgA in nasal and bronchoalveolar lavages and challenge with pVAXefa-1 reduced EHEC colonization in mice (Riquelme-Neira et al., 2015).

### Polysaccharide-based vaccines

Polysaccharides in conjugate vaccine against *Haemophilus influenzae* type b, pneumococcal and meningococcal bacteremia and meningitis have been successfully used for vaccine development. *E. coli* isolates produce two serotype-specific surface polysaccharides, namely lipopolysaccharide (LPS) O antigen and capsular polysaccharide K antigen. Variations in structures of these polysaccharides give rise to ~170 different O antigens and ~80 K antigens (Whitfield, 2006). Immunization with an O-specific polysaccharide of *E. coli* O157:H7, showed a significant increase of IgG against LPS. *E. coli* O157:H7 O-specific polysaccharide conjugated to recombinant exotoxin A of *P. aeruginosa* (O157-rEPA) administered to 2- to 5-year-old children showed that there was a >4-fold increase of IgG in serum after the first week of immunization. Serum anti-LPS IgG increased >8-fold at week 6 and 20-fold at week 26, when there was no difference among groups receiving one dose or two doses, but levels were >4-fold higher than in the pre-immune sera. The serum samples had antibacterial activity correlated with the IgG anti-LPS antibody titer. More importantly, vaccinated children showed mild to non-collateral reactions to the vaccine. This prospective vaccine seems to be a good candidate because of its safety and immune reactivity (Konadu et al., 1994, 1998, 1999; Ahmed et al., 2006).

### Improving the adjuvant effect

The suitability of an adjuvant is important in vaccine development to enhance the immunogenicity and reactivity of the antigens in the host immune system. Vaccination with EspB or the C-terminal of γ-intimin (280 amino acids, γ-IntC_280_) co-administered with the MALP-2 adjuvant (TLR6 agonist) enhanced IgG specific antibodies after a first intranasal immunization, whereas vaccination without MALP-2 increased IgG titers after the second boost (Cataldi et al., 2008). More recently it has been demonstrated that intranasal immunization of dams and later passive immunization of their offspring with either recombinant EspB or γ-intimin C280 plus MALP2 as adjuvant significantly reduced the intestinal colonization of bacteria and uremia level in serum (clinical parameter of the systemic effect of Shiga toxin) in the infant mice. In addition, both dams and suckling mice produced significant IgG titers and no renal or intestinal lesions were observed by histopathological examination (Rabinovitz et al., 2016).

The adjuvant effect has been also implemented by the combined expression of toxins and antigens at the same time, as a specific antigen combination. An example of this method was provided by the generation of a chimeric protein fusing Tir, Stx1B, Stx2B and the zonula occludens toxin (Zot). Intranasally immunized mice showed higher IgA and IgG response against the chimeric protein than subcutaneously immunized mice and reduced bacterial shedding in feces post-EHEC challenge. Comparison of the adjuvant effect of chimeric protein with or without Zot has showed greater with Zot, even though there were no IgG specific titers against it (Zhang et al., 2011).

### Future of EHEC vaccine development

EHEC infections and associated diseases are also related to other 6 STEC serotypes, O26:H11, O45:H2, O103:H11, O11, O121:H19, O145, other than the canonical serotypes O113:H21 and O157:H7. Treatment and prevention of infection, like vaccine development, should target these strains. More recently, new approaches have been implemented to find new antigens. For example, in an immunoproteomics analysis a Chilean group detected antigens with an immune reactive effect in patients infected with any of these STEC serotypes. They identified mainly outer membrane proteins, like OmpT and Cah, which were immune reactive with sera from HUS patients. The genes encoding these proteins are widely represented among *E. coli* pathotypes, but also in commensal strains. Other detected proteins included FliC, Ag43 (ETEC), NmpC, OmpF, OmpC, OmpA, Hek, EF-Tu, and L-asparaginase II (Montero et al., 2014).

## ETEC, the most studied InPEC for vaccine development

Enterotoxigenic *E. coli* is responsible for infection with traveler's diarrhea around the world and to date has been one of the most studied InPEC for vaccine targets. Children in developing countries are the most affected by ETEC infections and diarrheal diseases. Overall, it has been estimated that ETEC infections are the cause of 200 million diarrheal cases and between 170,000 and 380,000 deaths annually (Isidean et al., 2011; Chakraborty et al., 2015).

The symptoms of an ETEC infection include dysentery, headache, fever and vomiting. The infection can last up to 5 days, without specific treatment or antibiotics, but lethal cases are associated with children due to lack of immune protection and dehydration (Vidal et al., 2016). ETEC is transmitted by consuming contaminated water, food, and person to person contact, poor sanitation being one of the main factors in the pervasiveness of this pathogen in developing countries.

The main virulence factors associated with the pathogenesis of infection are colonization factor antigens (CFA), which are required for ETEC colonization and establishment in the gut, and heat-labile (LT) and heat-stable (ST) toxins, which are responsible for water and electrolyte discharge during infection (Vidal et al., 2016). Regarding the CF, they are mainly referred to as *E. coli* surface antigens (CS) composed by 26 characterized factors (CFA/I, CFA/III, CS2-CS26). These factors are not present at the same time in the ETEC strains, but can be carried in different combinations. Nonetheless, the presence of other non-fimbrial adhesins as EtpA, Tia, TibA, TleA, and EaeH, expressed in the prototypic strain H10407, has been described (Vidal et al., 2016). Concerning ETEC classification, the most common serotypes associated with ETEC are O6:H16 (LT/ST), O8:H9 (ST only), O25:NM (LT only), O78:H12 (ST only), O148:H28 (ST only), O153:H45 (ST only), and O169:H41 (ST only). However, serotypes isolated from different outbreaks do not necessarily belong to these serotypes or can differ in their prevalence. To date, isolated ETEC can be divided into 42 different clonal groups with a singular combination of CF and toxins (Croxen et al., 2013). Because ETEC is still a public health problem in developing countries, there is a big effort to develop a vaccine to prevent and reduce the incidence of infections caused by this pathotype.

### Toxin-based vaccines

The transcutaneous route has been used to immunize human volunteers with LT toxin delivered in a skin patch, a strategy designed to avoid the toxic effect of LT and which allows transfer of antigen from the skin to antigen-presenting Langerhans cells and then transport to draining lymph nodes. In this double-blind, placebo-controlled trial, human volunteers were immunized by applying the patches to the skin on the arms and then challenged with a LT/ETEC strain (McKenzie et al., 2007; Frech et al., 2008; Frerichs et al., 2008). As in previous reports, in 97–100% of cases, anti-LT IgG and IgA increased (4-fold). Although there was a delay in the onset of disease, this strategy did not prevent the illness after challenge (McKenzie et al., 2007; Frech et al., 2008). The later refinement of the scale of disease parameters and correlation between symptoms and signs after human challenge enabled classification of specific patch vaccines with no efficacy (Porter et al., 2016).

The ST subunit A (STa) is known to be poorly immunogenic as an antigen for vaccine use, however, its immunogenicity can be enhanced by combination with stronger antigens, like LT or other adjuvants, and by reducing its toxicity by changing essential amino acids. Mice immunized with a STa_P13F_-LT_R192G_ toxoid fusion protein were able to develop specific IgG antibodies for LT and STa proteins in serum and feces and only IgA in feces. In addition, the STa and LT antibodies from fecal samples reduced cGMP and cAMP, respectively, in T-84 culture cells. This study indicates that the STaP13F toxoid is immunogenic when fused to LT toxoid and elicited neutralizing antitoxin antibodies. These findings will be useful for developing safe and effective toxoid vaccines linked to ETEC-STa-producing strains (Liu et al., 2011).

### Autotransporters

Immunoproteomic analysis has also been employed as a strategy for the identification of antigens able to generate an immune response in the host during the process of infection. For this type of approach, reacting human or mice sera obtained after ETEC infections enabled identification of immune reactive molecules in culture supernatant, outer membrane, and outer membrane vesicle preparations by matrix-assisted laser desorption ionization–time of flight mass spectrometry (MALDI-TOF MS). In this analysis, hypothetical proteins homologous to other pathovars (UPEC, APEC, Crohn's disease-associated isolates) and pathogens (*Vibrio cholerae* and group A *streptococci*) were identified, suggesting their role in the pathogenesis. In addition, autotransporters (AT) such as EtpA, Antigen 43 and TibA were also found to be reactive during the infection process (Roy et al., 2010).

*In silico* tools have been used to identify other remarkable virulence factors as immunogenic molecules. From those studies, autotransporters, which are important in biofilm formation, such as pAT, antigen 43 and EatA, have been identified exclusively in ETEC or other pathotypes, but are absent in commensal strains. Recombinant Ag43 and pAT autotransporters increased fecal IgA and provided relative protection against ETEC intestinal colonization in immunized mice. Interestingly, these autotransporters are also recognized by sera from patients with ETEC diarrhea, confirming their expression during ETEC infections (Harris et al., 2011).

### Adhesins

Adhesins have been widely employed as target vaccines as several of them are considered to be virulence factors with an important role in colonization and bacterial pathogenesis.

The ETEC two-partner secretion protein A (EtpA) is an encoded plasmid protein and secreted non-fimbrial adhesin. When mice were immunized with the recombinant fully glycosylated form of this protein, EtpA inhibited the colonization of ETEC wild-type strains. Also, EtpA antibodies present in the sera of mice immunized with the glycoprotein enhanced by flagellin inhibited colonization in *in vitro* assays (Roy et al., 2009). In addition, a study of EtpA and CexE in flagellin-free OMV showed that these antigens were able to reduce wild-type ETEC colonization in mice without the flagellin contribution (Roy et al., 2011).

### Attenuated bacteria platforms

Some studies have shown that strain ETEC E1392/75-2A confers protection in 75% of human volunteers immunized and challenged with ETEC wild-type strains. However, this strain induced mild diarrhea in 15% of volunteers. By deleting different genes in the chromosomes it was possible to attenuate the strain and also to reduce its reactogenicity. Two different mutant strains were generated and attenuated by mutating aroC, ompR genes (PTL-002 mutant) and aroC, ompC and ompF genes (PTL-003 mutant). Note that both of these mutants express CFA/II: CS1, CS3 and are ST/LT spontaneously mutated. These mutants were first tested in a mouse model, and further tested in human volunteers, in whom adverse effects were reduced. Interestingly, the strain PTL-003 showed higher immunogenicity provided by increased IgA and IgG levels and antibody response to CS1 and CS3. However, poor protection in subjects orally immunized was obtained and only reduced diarrheal manifestations after challenge with wild-type ETEC E24377A (Turner et al., 2001; McKenzie et al., 2006, 2008).

The MEV or Etvax vaccine is another cocktail of four inactivated recombinant bacteria expressing separately different CFs, in which CS6 is expressed in a K12 strain and CFA/I, CS3, CS5 in an ETEC O78 toxin-negative strain. Formalin- or phenol-attenuated bacteria were used in preclinical and clinical phase I studies (Lundgren et al., 2014) and immunized together with an LCTBA hybrid protein (which has seven amino acids in CTB replaced by corresponding amino acids of LTB) and a double mutant LT (dmLT, which has two replacements in the A subunit, LTR192G/L211A (Norton et al., 2011), eliminating the toxic effect and leaving the adjuvant effect of the LT) (Lundgren et al., 2014). In preclinical studies, they showed immunoreactivity in mice, resulting in high titers of fecal, jejunal tissue extracts and serum IgA and IgG, enhanced by the presence of dmLT (Holmgren et al., 2013). Furthermore, a double-blind, randomized, placebo-controlled phase I trial confirmed increasing titers of IgA and IgG in sera and fecal samples in humans. Volunteers orally immunized with the same vaccine but different amounts of dmLT (10 or 25 μg) showed significant increases in intestinal, fecal and serum IgA. Also, humans immunized with the formulation using 10 μg of dmLT showed a significant response to all the antigens, but an even higher response to CS6. Additionally, the vaccine combination was well tolerated and considered to be safe for human use (Holmgren et al., 2013; Lundgren et al., 2014).

Attenuated bacterial strategies for vaccine development have included the use of different bacterial species. Non-pathogenic and *V. cholerae* strains were engineered to express CFA/I operon (cfaA-B-C-E) and killed by formalin to immunize mice orally with CT as adjuvant. The results showed increased IgA and IgG+IgM specific antibody titers in serum against CFA/I. Antigen delivery was thought to be favored by the fact that *V. cholerae* can attach to M cells, probably enhancing bacterial attachment to the intestinal mucosal by the overexpression of CFA/I. Increasing titers of IgA and IgG+M were found against *V. cholerae* O1 LPS after immunization (Tobias et al., 2008). When CS2 was added to formalin-killed *E. coli* K12 over-expressing CFA/I and administered orally, a similar immune response (increase in IgG+M in sera and IgA in feces) against *cfaB* from CFA/I and *cotA* from CS2 was observed (Tobias et al., 2010).

Following the same strategy as the PTL attenuated vaccines, mentioned above, three other ETEC strains were attenuated by the same gene mutations and used as prospective vaccines. These strains expressed diverse CFA elements including CFA/I (ACAM2010 strain), CS2 and CS3 (ACAM2007 strain) and CS1, CS2, and CS3 (ACAM2017 strain). As shown by PTL-003, mentioned above, there was an immune response against those CFA elements and the IgA level increased in orally immunized human volunteers. However, the inflammatory interleukins IL-6 and IL-8 were not detected (Daley et al., 2007).

CS21, an adhesion factor belonging to the Class-B Type 4 pili as BFP and TCP, was tested as a potential vaccine candidate. CS21 including its major subunit LngA conferred a certain immunoreactivity in a mouse model. The immunogenicity of CS21 and LngA in different adjuvant formulations showed that CS21/LngA combined with cholera toxin and administered intraperitoneally increased specific IgG antibodies in serum and IgA antibody levels in fecal and intestinal lavages. Interestingly, anti-LngA antibodies were also raised following intraperitoneal administration of CS21 formulated with incomplete Freund's adjuvant and after challenge in mice this formulation stopped bacterial shedding. By contrast, CS21 plus CT as adjuvant was more effective when administered intranasally. This study supports the idea of using CF as a promising antigen and shows that administration routes play an important role in eliciting an effective immune response and protection (Zhang et al., 2016).

Other colonization factors from different ETEC strains have been studied as vaccine candidates, mainly because there is a usual combination of co-expression of these factors in ETEC strains, making it a hallmark to distinguish the different strains. To cover a wide range of ETEC strain variability involved in diarrheal infections, a non-toxigenic *E. coli* was modified to express every CS2, CS4, CS5, or CS6 independently or co-expressed with CFA/I. Immunization of mice with recombinant bacteria co-expressing CFA/I and CS2 induced IgG+IgM antibodies in sera and IgA in fecal samples. The antibody titers for recombinant bacteria were higher than for wild-type stains. CS6 overexpressed in a non-toxigenic strain, and used as a killed vaccine, increased IgG+IgM antibodies in sera and fecal IgA antibodies like the other strains (Tobias et al., 2010, 2011). Although CS6 was previously reported as a weak antigen in inducing the immune response, this study improved its properties as a vaccine candidate.

More recently, the generation of three strains in the ACE527 ETEC complex has been the most promising vaccine in phase II studies. Strains in the ACE527 were attenuated, made antibiotic-sensitive, and genetically engineered to overexpress different colonization factors. All of them were attenuated by mutation in *aroC, ompC and ompF*, and were safe and immunogenic in humans as PTL-003 attenuated strain (Turner et al., 2001). These three strains ACAM2022 (O141:H5, expressing CS5 and CS6), ACAM2025 (O39:H12, expressing CFA/I) and ACAM2027 (O71:H-, expressing CS2, CS3, and CS1) also express the heat-labile toxin pentamer B subunits. In particular, the LTB gene encoding heat-labile toxin pentamer B was inserted in the ACAM2022 genome to ensure its stability and avoid its loss during vaccination. In addition, the gene encoding CS1 was inserted in the ACAM2027 genome by replacing *ompC*. Although other CFs, as CS3, CS5m CS6 and CFA/I, were maintained in their native plasmid, they were stable (Turner et al., 2011).

Human clinical trials, testing two different oral doses 3 weeks apart of ACE527 (doses of 10^11^ or 3 × 10^10^ CFU each strain) in a CeraVacx buffer, showed that IgA and IgG increased in serum, for LTB, CFA/I, CS3, CS5, and CS6. After immunization, subjects were challenged with the ETEC H10407 wild-type strain. As a result, vaccination helped shorten the duration of diarrhea and reduced shedding of the wild-type strain, which was up to 20-fold less than in control groups 2 days after challenge, and conferred ranging 33 and 98% protection against the wild-type strain. Bacterial shedding decreased even more in re-challenged subjects (Harro et al., 2011a,b). Overall, this ACE527 formulated in CeraVax reduced diarrhea by 29% and showed 26.5% efficacy (Harro et al., 2011a; Darsley et al., 2012; Porter et al., 2016). When the ACE527 vaccine combination was formulated with LTR192GL211A adjuvant, efficacy increased to 50% (Porter et al., 2016).

### Outer membrane vesicles

Outer membrane vesicles have been largely employed as an antigen delivery system. The expression of heterologous surface antigens has been applied not only to amplify the protection against different pathogens, but also to increase the antigenicity of such antigens. Recently, this system was used in two different ways. The first one used an ETEC mutated in *msbB* (lipid A acyltransferase) and *eltA* (labile toxin subunit A) to decrease the OMV toxicity level. The OMV generated from this mutant strain (EΔ*msbB*Δ*eltA*) were used to immunize adult mice and test immunoreactivity. It was shown that the detoxified OMV yielded higher titers of IgG1, IgM, and IgA, in comparison to the OMV from a wild-type strain. Later, offspring born to dams immunized with OMV-EΔ*msbB*Δ*eltA*, and passively immunized by suckling and then challenged with ETEC displayed low colonization levels. In the second part of this approach, ETEC antigens including the adhesins FliC and CFA/I were expressed as heterologous proteins in *V. cholerae* OMV. This strain was engineered by deletion of *msbB, ctxAB* (cholera toxin subunits A and B) and *flaA* (major flagellin A) (VΔ*msbB*Δ*ctxAB*Δ*flaA*). As in the previous test with the OMV from ETEC EΔ*msbB*Δ*eltA*, the immune response against VΔ*msbB*Δ*ctxAB*Δ*flaA* expressing FliC-CFA/I displayed the same pattern in adult mice and a similar colonization level in the neonatal mouse model. While there was an immune response against both heterologously expressed ETEC antigens in *V. cholerae*, none of these approaches resulted in improved protection (Leitner et al., 2015).

## Immune protection for EPEC infections

Enteropathogenic *E. coli*, non-invasive bacteria colonize the small intestine, causing moderate to acute diarrhea mainly in children under 2 years old in developing countries. The peculiar characteristic of this pathogen and pathotype is the presence of the LEE pathogenic genomic island that encodes virulence factors, such as intimin and the translocated intimin receptor (Tir), associated with the T3aSS necessary to produce A/E lesions on the intestinal microvilli. A/E lesions consist in the rearrangement of actin and tight attachment of bacteria to host cells by translocation of effectors. Tir, which as a receptor for intimin and promotes actin rearrangement, is one of the main proteins translocated from the bacterium to the eukaryotic cell through the T3aSS. In contrast to LEE-positive EHEC, intimin and Tir are important for the adhesion and establishment of EPEC in eukaryotic cells. Also, it is well known that EPEC has a capacity for localized adherence produced by a bundle-forming pilus (BFP, BfpA being its major subunit). This pilus is encoded by the plasmid *E. coli* adherence factor (pEAE) (Ochoa et al., 2008). Nonetheless, this pathotype has been subcategorized in either (i) typical EPEC (tEPEC) carrying the EPEC adherence factor plasmid (pEAF) and LEE island, or (ii) atypical EPEC (aEPEC), which lack the pEAF (Croxen et al., 2013; Gomes et al., 2016; Scaletsky and Fagundes-Neto, 2016). In the case of the aEPEC, which lacks the BFP, it has been shown that they can produce an LA-like pattern promoted by intimin (subtype omicron), and in some cases the aggregative or diffuse patterns (Hernandes et al., 2008; Gomes et al., 2016).

The most common O serogroups for the classic EPEC are: O26, O39, O55, O86, O88, O103, O111, O114, O119, O125ac, O126, O127, O128ab, O142, O145, O157, and O158. On the other hand, even if the most recurrent aEPEC serogroups are O51, O145, O26, O55, and O111, many others do not belong to the same tEPEC serogroups and some are neither O nor H typeable (Hernandes et al., 2009; Hu and Torres, 2015). tEPEC is most commonly recovered from humans, while aEPECs are recovered from farmed and domestic animals and are considered as a zoonotic pathogen (Hernandes et al., 2009; Gomes et al., 2016). However, the latest reports have shown an increased emergence of aEPECs in developed and developing countries (Ochoa and Contreras, 2011; Ingle et al., 2016).

The incidence and prevalence of EPEC infections vary according to diagnostic methods. Molecular methods targeting specific genes (as intimin) seem to show an incidence of 5–10% of pediatric diarrheal cases in developing countries, whereas diagnosis by Hep-2 adherence-pattern and serotyping has shown average prevalence rates of 10–20% (Ochoa et al., 2008). More recently, it has been shown that aEPECs are more prevalent than tEPEC in both developing and developed countries (Ochoa et al., 2008).

### Passive immunization against EPEC

Natural immune protection after initial EPEC infection has been observed and antibodies can protect against future infections. Breast feeding has an important role in natural immune protection against EPEC infections. Studies in developing countries have shown that IgA antibodies, mainly against intimin, EspA, EspB, EspD, EspC, and BFP, can be transmitted by colostrum from mothers to their infants. These antibodies can prime and protect the neonates in the first hours of birth against EPEC (Parissi-Crivelli et al., 2000; Noguera-Obenza et al., 2003; Durand et al., 2013). In addition, EspA, B, C, and D immune responses might confer cross-protection against EHEC strains that carry the LEE locus. IgA antibodies against BfpA and EspB have been detected in stool specimens from breastfed infants with acute diarrhea, but are absent in healthy children and non-breastfed infants (Quintana Flores et al., 2002). The prevalence of anti-BfpA fecal antibodies has indicated the immunogenicity of BfpA, but their functionality as blocking or neutralizing antibodies has not been fully elucidated (de Souza Campos Fernandes et al., 2003).

Among candidates considered for EPEC vaccine development, EspB is one of the most important proteins for EPEC pathogenesis as it contributes to A/E lesions in epithelial cells, helps Tir phosphorylation and entrance into host cells, and mediates anti-phagocytosis by inhibiting myosin function (Taylor et al., 1998; Iizumi et al., 2007). The role of EspB in virulence has been studied in a human clinical trial comparing the ability to cause diarrhea of an isogenic EPEC mutant lacking EspB versus a wild-type strain. Diarrhea developed in 9 of 10 volunteers who ingested the wild-type strain, but in only 1 of 10 volunteers who ingested the EspB mutant strain. In addition, a biopsy performed in two volunteers infected with the mutant showed no destruction of the microvillus brush border (Tacket et al., 2000).

Vaccine development is made trickier by the existence of three variants of EspB, i.e., α, β, and γ, where the α variant is subdivided into 1, 2, and 3 but without clear correlation between an EspB protein subtype and a specific serogroup of EPEC and EHEC. A recent report describes the production of a hybrid recombinant EspB toxin that comprises all known variants of the protein. This recombinant protein has been proposed as an antigen for the production of antibodies with broad-range detection of EspB-bearing bacteria (Caetano et al., 2017). Furthermore, phage display library screening has identified a synthetic peptide (YFPYSHTSPRQP) able to bind EspB. These candidates significantly decreased by up to 40% the adherence of EPEC to cultures of HEp-2 cells (Li et al., 2016). Other examples of vaccine candidates specific for EPEC and LEE positive strains have used the translocator protein EspA as target. Synthetic peptides (CoilA [LTTTVNN][SQLEIQQ]M and CoilB [MSNTLNL][LTSARSD]M) representing coiled-coil regions of EspA inhibited EspA and consequently T3SS assembly. These peptides effectively inhibited T3SS-dependent hemolysis of red blood cells by the EPEC E2348/69 strain, reduced actin pedestal formation in HEp-2 cells, and impaired T3SS-mediated protein translocation into epithelial cells (Larzabal et al., 2010). Overall, peptide-based strategies could prove effective in blocking EPEC and EHEC infections, albeit while restricting their use to the development of vaccines only against LEE positive strains.

So far we have no vaccine to prevent EPEC infection, which occurs mainly in developing countries and causes acute diarrhea, which is still a public health concern. The most recent information obtained from both genome sequencing and maternal/passive immunization will enhance the search for novel antigens to be used for vaccine development (Torres, 2017b).

## Conclusions and future perspectives

One of today's major public health problems is the burden of diarrheal diseases caused by several etiological agents and for which preventive strategies such as vaccines to block infections do not exist. In this scenario, InPECs contribute significantly to the burden of diseases, especially in low- and middle-income countries. In the last years many efforts have been made to identify efficacious vaccines able to prevent diarrheal disease and reduce the burden worldwide.

Recently, with the advent of new technologies, the field of vaccinology has changed dramatically. Whole-genome sequencing together with powerful bioinformatics tools to analyze the genomes have contributed to the understanding of the *E. coli* biology and its high genomic plasticity. *E. coli* strains share a common core genome which is highly conserved among all strains, while the encoding genes related to diverse pathogenic functions involved in infection, disease development and host-pathogen interaction vary among pathotypes. Moreover, the presence of different virulence factors in different *E. coli* phylogenetic groups and the different clinical outcomes (severe, lethal, non-lethal or asymptomatic diarrhea) of the infection, suggests that the bacterium can use several strategies to cause disease, with virulence associated genes that can be acquired and/or lost and many times. The high genome and antigen diversity of *E. coli* has been one of the main factors hampering the development of effective treatments against the various diseases caused by *E. coli* infection. Approaches like Reverse Vaccinology, based on the scanning of the annotated complete pathogen genome and bioinformatic prediction of the most likely vaccines candidates, have enabled the identification of promising conserved antigen that could be the basis for the development of safe and broadly protective vaccines against pathogenic *E. coli* (Moriel et al., 2016).

Overall, as illustrated in this review, a number of different strategies have been used for the identification of promising antigens present and conserved in different InPEC pathotypes. A new vaccine should be able to prevent diarrheal diseases caused not only by ETEC or EHEC infections, but also by other pathotypes that are becoming even more relevant by the epidemiology and from a public health point of views. Importantly, treatments and preventive interventions should also target non-O157 EHEC serotype, and other pathotypes as DAEC, EIEC, EAEC, for which vaccine development and discovery studies are scarce or nonexistent (Bouzari et al., 2010).

Recently, it has been proposed that multidisciplinary research, collaboration and partnerships should adopt the One Health concept, as done in Latin America (Torres, 2017a). In particular, this concept states that there is a interdependence between human health and the environment, animals and human beings. To achieve this objective, research should be extended to animal pathogenic strains. Examples of this are *E. coli* research studies in animal reservoirs (Etcheverría et al., 2016).

Although the vaccine field is advancing very fast, and new sophisticated approaches are being applied for the identification of new and effective antigens, the battle against InPEC infections is still open and only multidisciplinary research efforts in the field of microbiology, immunology, epidemiology, medicine, clinical, veterinary and public health, could allow to understand the disease in different settings and the design of new preventive strategies (CDC)^1^.

## Author contributions

MR-L and RR: wrote the manuscript; MR-L, RM, MP, MD, and RR: contributed to the preparation of the manuscript and to the ideas and concepts it contains. All authors reviewed and approved the manuscript. The authors are grateful to David Marsh (djmarsh@wanadoo.fr) for correcting the English of the manuscript.

### Conflict of interest statement

RR and MP are permanent employees of GSK. The authors declare that GSK provided support in the form of salaries. MR-L and RM participated in a postgraduate studentship program at GSK/INRA.

## Correction Note

A correction has been made to this article. Details can be found at: 10.3389/fmicb.2025.1679236.

## Footnotes

^1^CDC. Available: www.cdc.gov/onehealth/basics/index.html.

## References

[B1] Ahmed A. Li J. Shiloach Y. Robbins J. B. Szu S. C. (2006). Safety and immunogenicity of *Escherichia coli* O157 O-specific polysaccharide conjugate vaccine in 2-5-year-old children. J. Infect. Dis. 193, 515–521. 10.1086/499821 16425130

[B2] Amani J. Mousavi S. L. Rafati S. Salmanian A. H. (2009). *In silico* analysis of chimeric espA, eae and tir fragments of *Escherichia coli* O157:H7 for oral immunogenic applications. Theor. Biol. Med. Model. 6:28. 10.1186/1742-4682-6-28 19995413 PMC3224939

[B3] Amani J. Mousavi S. L. Rafati S. Salmanian A. H. (2011). Immunogenicity of a plant-derived edible chimeric EspA, Intimin and Tir of *Escherichia coli* O157:H7 in mice. Plant Sci. 180, 620–627. 10.1016/j.plantsci.2011.01.004 21421410

[B4] Amani J. Salmanian A. H. Rafati S. Mousavi S. L. (2010). Immunogenic properties of chimeric protein from espA, eae and tir genes of *Escherichia coli* O157: H7. Vaccine 28, 6923–6929. 10.1016/j.vaccine.2010.07.061 20709010

[B5] Bentancor L. V. Bilen M. Brando R. J. Ramos M. V. Ferreira L. C. Ghiringhelli P. D.. (2009). A DNA vaccine encoding the enterohemorragic *Escherichia coli* Shiga-like toxin 2 A2 and B subunits confers protective immunity to Shiga toxin challenge in the murine model. Clin. Vaccine Immunol. 16, 712–718. 10.1128/CVI.00328-08 19176691 PMC2681575

[B6] Bitzan M. Poole R. Mehran M. Sicard E. Brockus C. Thuning-Roberson C.. (2009). Safety and pharmacokinetics of chimeric anti-Shiga toxin 1 and anti-Shiga toxin 2 monoclonal antibodies in healthy volunteers. Antimicrob. Agents Chemother. 53, 3081–3087. 10.1128/AAC.01661-08 19414580 PMC2704659

[B7] Bouzari S. Dashti A. Jafari A. Oloomi M. (2010). Immune response against adhesins of enteroaggregative *Escherichia coli* immunized by three different vaccination strategies (DNA/DNA, Protein/Protein, and DNA/Protein) in mice. Comp. Immunol. Microbiol. Infect. Dis. 33, 215–225. 10.1016/j.cimid.2008.10.002 19022502

[B8] Brzuszkiewicz E. Thürmer A. Schuldes J. Leimbach A. Liesegang H. Meyer F. D.. (2011). Genome sequence analyses of two isolates from the recent *Escherichia coli* outbreak in Germany reveal the emergence of a new pathotype: entero-Aggregative-Haemorrhagic *Escherichia coli* (EAHEC). Arch. Microbiol. 193, 883–891. 10.1007/s00203-011-0725-6 21713444 PMC3219860

[B9] Caetano B. A. Rocha L. B. Carvalho E. Piazza R. M. F. Luz D. (2017). Immunogenic domains and secondary structure of *Escherichia coli* recombinant secreted protein *Escherichia coli*-secreted protein, B. Front. Immunol. 8:477. 10.3389/fimmu.2017.00477 28484467 PMC5402224

[B10] Cai K. Gao X. Li T. Hou X. Wang Q. Liu H.. (2010). Intragastric immunization of mice with enterohemorrhagic *Escherichia coli* O157:H7 bacterial ghosts reduces mortality and shedding and induces a Th2-type dominated mixed immune response. Can. J. Microbiol. 56, 389–398. 10.1139/W10-025 20555401

[B11] Cai K. Gao X. Li T. Wang Q. Hou X. Tu W.. (2011). Enhanced immunogenicity of a novel Stx2Am-Stx1B fusion protein in a mice model of enterohemorrhagic *Escherichia coli* O157:H7 infection. Vaccine 29, 946–952. 10.1016/j.vaccine.2010.11.035 21134452

[B12] Cai K. Tu W. Liu Y. Li T. Wang H. (2015). Novel fusion antigen displayed-bacterial ghosts vaccine candidate against infection of *Escherichia coli* O157:H7. Sci. Rep. 5:17479. 10.1038/srep17479 26626573 PMC4667225

[B13] Calderon Toledo C. Arvidsson I. Karpman D. (2011). Cross-reactive protection against enterohemorrhagic *Escherichia coli* infection by enteropathogenic *E. coli* in a mouse model. Infect. Immun. 79, 2224–2233. 10.1128/IAI.01024-10 21402761 PMC3125830

[B14] Cataldi A. Yevsa T. Vilte D. A. Schulze K. Castro-Parodi M. Larzábal M.. (2008). Efficient immune responses against Intimin and EspB of enterohaemorragic *Escherichia coli* after intranasal vaccination using the TLR2/6 agonist MALP-2 as adjuvant. Vaccine 26, 5662–5667. 10.1016/j.vaccine.2008.07.027 18675866

[B15] Chakraborty S. Harro C. DeNearing B. Ram M. Feller A. Cage A.. (2015). Characterization of mucosal immune responses to enterotoxigenic *Escherichia coli* vaccine antigens in a human challenge model: response profiles after primary infection and homologous rechallenge with strain H10407. Clin. Vaccine Immunol. 23, 55–64. 10.1128/CVI.00617-15 26581889 PMC4711095

[B16] Cheng Y. Feng Y. Luo P. Gu J. Yu S. Zhang W. J.. (2009). Fusion expression and immunogenicity of EHEC EspA-Stx2Al protein: implications for the vaccine development. J. Microbiol. 47, 498–505. 10.1007/s12275-009-0116-8 19763426

[B17] Coombes B. K. Gilmour M. W. Goodman C. D. (2011). The evolution of virulence in non-o157 shiga toxin-producing *Escherichia coli*. Front. Microbiol. 2:90. 10.3389/fmicb.2011.00090 21833329 PMC3153049

[B18] Croxen M. A. Finlay B. B. (2010). Molecular mechanisms of *Escherichia coli* pathogenicity. Nat. Rev. Microbiol. 8, 26–38. 10.1038/nrmicro2265 19966814

[B19] Crim S. M. Griffin P. M. Tauxe R. Marder E. P. Gilliss D. Cronquist A. B. Henao.. (2015). Preliminary Incidence and Trends of Infection with Pathogens Transmitted Commonly through Food — Foodborne Diseases Active Surveillance Network, 10 U.S. Sites, 2006–2014. MMWR Morbidity and Mortality Weekly Report. Atlanta, GA: Center for Surveillance, Epidemiology, and Laboratory Services, Centers for Disease Control and Prevention (CDC). PMC4584825 25974634

[B20] Croxen M. A. Law R. J. Scholz R. Keeney K. M. Wlodarska M. Finlay B. B. (2013). Recent advances in understanding enteric pathogenic *Escherichia coli*. Clin. Microbiol. Rev. 26, 822–880. 10.1128/CMR.00022-13 24092857 PMC3811233

[B21] Daley A. Randall R. Darsley M. Choudhry N. Thomas N. Sanderson I. R.. (2007). Genetically modified enterotoxigenic *Escherichia coli* vaccines induce mucosal immune responses without inflammation. Gut 56, 1550–1556. 10.1136/gut.2006.112805 17566016 PMC2095642

[B22] Darsley M. J. Chakraborty S. DeNearing B. Sack D. A. Feller A. Buchwaldt C.. (2012). The oral, live attenuated enterotoxigenic *Escherichia coli* vaccine ACE527 reduces the incidence and severity of diarrhea in a human challenge model of diarrheal disease. Clin. Vaccine Immunol. 19, 1921–1931. 10.1128/CVI.00364-12 23035175 PMC3535858

[B23] De Gregorio E. Rappuoli R. (2012). Vaccines for the future: learning from human immunology. Microb. Biotechnol. 5, 149–155. 10.1111/j.1751-7915.2011.00276.x 21880117 PMC3815775

[B24] de Souza Campos Fernandes R. C. Quintana Flores V. M. Sousa de Macedo Z. Medina-Acosta E. (2003). Coproantibodies to the enteropathogenic *Escherichia coli* vaccine candidates BfpA and EspB in breastfed and artificially fed children. Vaccine 21, 1725–1731. 10.1016/S0264-410X(02)00525-X 12639496

[B25] Durand D. Ochoa T. J. Bellomo S. M. Contreras C. A. Bustamante V. H. Ruiz J.. (2013). Detection of secretory immunoglobulin A in human colostrum as mucosal immune response against proteins of the Type III secretion system of *Salmonella, Shigella* and enteropathogenic *Escherichia coli*. Pediatr. Infect. Dis. J. 32, 1122–1126. 10.1097/INF.0b013e318293306c 23538526 PMC3776007

[B26] Etcheverría A. I. Lucchesi P. M. A. Krüger A. Bentancor A. B. Padola N. L. (2016). *Escherichia coli* in Animals, in Escherichia coli in the Americas, ed Torres A. G. (Cham: Springer International Publishing), 149–172.

[B27] Feavers I. M. Maiden M. C. J. (2017). Recent progress in the prevention of serogroup b meningococcal disease. Clin. Vaccine Immunol. 24:e00566–16. 10.1128/CVI.00566-16 28356256 PMC5424234

[B28] Foster M. A. Iqbal J. Zhang C. McHenry R. Cleveland B. E. Romero-Herazo Y.. (2015). Enteropathogenic and enteroaggregative *E. coli* in stools of children with acute gastroenteritis in Davidson County, Tennessee. Diagn. Microbiol. Infect. Dis. 83, 319–324. 10.1016/j.diagmicrobio.2015.07.016 26298817 PMC4618545

[B29] Frank C. Werber D. Cramer J. P. Askar M. Faber M. an der Heiden M.. (2011). Epidemic profile of Shiga-toxin-producing *Escherichia coli* O104:H4 outbreak in Germany. N. Engl. J. Med. 365, 1771–1780. 10.1056/NEJMoa1106483 21696328

[B30] Frech S. A. Dupont H. L. Bourgeois A. L. McKenzie R. Belkind-Gerson J. Figueroa J. F.. (2008). Use of a patch containing heat-labile toxin from *Escherichia coli* against travellers' diarrhoea: a phase II, randomised, double-blind, placebo-controlled field trial. Lancet 371, 2019–2025. 10.1016/S0140-6736(08)60839-9 18554712

[B31] Frerichs D. M. Ellingsworth L. R. Frech S. A. Flyer D. C. Villar C. P. Yu J.. (2008). Controlled, single-step, stratum corneum disruption as a pretreatment for immunization via a patch. Vaccine 26, 2782–2787. 10.1016/j.vaccine.2008.02.070 18455283

[B32] Fujii J. Naito M. Yutsudo T. Matsumoto S. Heatherly D. P. Yamada T.. (2012). Protection by a recombinant *Mycobacterium bovis* Bacillus Calmette-Guerin vaccine expressing Shiga toxin 2 B subunit against Shiga toxin-producing *Escherichia coli* in mice. Clin. Vaccine Immunol. 19, 1932–1937. 10.1128/CVI.00473-12 23035176 PMC3535872

[B33] Gansheroff L. J. Wachtel M. R. O'Brien A. D. (1999). Decreased adherence of enterohemorrhagic *Escherichia coli* to HEp-2 cells in the presence of antibodies that recognize the C-terminal region of intimin. Infect. Immun. 67, 6409–6417. 10569757 10.1128/iai.67.12.6409-6417.1999 PMC97049

[B34] Gao X. Cai K. Li T. Wang Q. Hou X. Tian R.. (2011). Novel fusion protein protects against adherence and toxicity of enterohemorrhagic *Escherichia coli* O157:H7 in mice. Vaccine 29, 6656–6663. 10.1016/j.vaccine.2011.06.106 21742003

[B35] Gao X. Cai K. Shi J. Liu H. Hou X. Tu W.. (2009). Immunogenicity of a novel Stx2B-Stx1B fusion protein in a mice model of Enterohemorrhagic *Escherichia coli* O157:H7 infection. Vaccine 27, 2070–2076. 10.1016/j.vaccine.2009.01.115 19428832

[B36] García-Angulo V. A. Kalita A. Kalita M. Lozano L. Torres A. G. (2014). Comparative genomics and immunoinformatics approach for the identification of vaccine candidates for enterohemorrhagic *Escherichia coli* O157:H7. Infect. Immun. 82, 2016–2026. 10.1128/IAI.01437-13 24595137 PMC3993450

[B37] Garmendia J. Frankel G. Crepin V. F. (2005). Enteropathogenic and enterohemorrhagic *Escherichia coli* infections: translocation, translocation, translocation. Infect. Immun. 73, 2573–2585. 10.1128/IAI.73.5.2573-2585.2005 15845459 PMC1087358

[B38] Giuliani M. M. Adu-Bobie J. Comanducci M. Aricò B. Savino S. Santini L.. (2006). A universal vaccine for serogroup B meningococcus. Proc. Natl. Acad. Sci. U.S.A. 103, 10834–10839. 10.1073/pnas.0603940103 16825336 PMC2047628

[B39] Goldwater P. N. Bettelheim K. A. (2012). Treatment of enterohemorrhagic *Escherichia coli* (EHEC) infection and hemolytic uremic syndrome (HUS). BMC Med. 10:12. 10.1186/1741-7015-10-12 22300510 PMC3286370

[B40] Gomes T. A. T. Yamamoto D. Vieira M. A. M. Hernandes R. T. (2016). Atypical Enteropathogenic *Escherichia coli*, in Escherichia coli in the Americas, ed Torres A. G.. (Cham: Springer International Publishing), 77–96.

[B41] Gu J. Ning Y. Wang H. Xiao D. Tang B. Luo P.. (2011). Vaccination of attenuated EIS-producing *Salmonella* induces protective immunity against enterohemorrhagic *Escherichia coli* in mice. Vaccine 29, 7395–7403. 10.1016/j.vaccine.2011.07.069 21807051

[B42] Harris J. A. Roy K. Woo-Rasberry V. Hamilton D. J. Kansal R. Qadri F.. (2011). Directed evaluation of enterotoxigenic *Escherichia coli* autotransporter proteins as putative vaccine candidates. PLoS Negl. Trop. Dis. 5:e1428. 10.1371/journal.pntd.0001428 22163060 PMC3232201

[B43] Harro C. Chakraborty S. Feller A. DeNearing B. Cage A. Ram M.. (2011a). Refinement of a human challenge model for evaluation of enterotoxigenic *Escherichia coli* vaccines. Clin. Vaccine Immunol. 18, 1719–1727. 10.1128/CVI.05194-11 21852546 PMC3187035

[B44] Harro C. Sack D. Bourgeois A. L. Walker R. DeNearing B. Feller A.. (2011b). A combination vaccine consisting of three live attenuated enterotoxigenic *Escherichia coli* strains expressing a range of colonization factors and heat-labile toxin subunit B is well tolerated and immunogenic in a placebo-controlled double-blind phase I trial in healthy adults. Clin. Vaccine Immunol. 18, 2118–2127. 10.1128/CVI.05342-11 21994354 PMC3232709

[B45] Hernandes R. T. Elias W. P. Vieira M. A. Gomes T. A. (2009). An overview of atypical enteropathogenic *Escherichia coli*. FEMS Microbiol. Lett. 297, 137–149. 10.1111/j.1574-6968.2009.01664.x 19527295

[B46] Hernandes R. T. Silva R. M. Carneiro S. M. Salvador F. A. Fernandes M. C. Padovan A. C.. (2008). The localized adherence pattern of an atypical enteropathogenic *Escherichia coli* is mediated by intimin omicron and unexpectedly promotes HeLa cell invasion. Cell. Microbiol. 10, 415–425. 10.1111/j.1462-5822.2007.01054.x 17910741

[B47] Holmgren J. Bourgeois L. Carlin N. Clements J. Gustafsson B. Lundgren A.. (2013). Development and preclinical evaluation of safety and immunogenicity of an oral ETEC vaccine containing inactivated *E. coli* bacteria overexpressing colonization factors CFA/I, CS3, CS5 and CS6 combined with a hybrid LT/CT B subunit antigen, administered alone and together with dmLT adjuvant. Vaccine 31, 2457–2464. 10.1016/j.vaccine.2013.03.027 23541621

[B48] Hu J. Torres A. G. (2015). Enteropathogenic *Escherichia coli*: foe or innocent bystander? Clin. Microbiol. Infect. 21, 729–734. 10.1016/j.cmi.2015.01.015 25726041 PMC4497942

[B49] Iizumi Y. Sagara H. Kabe Y. Azuma M. Kume K. Ogawa M.. (2007). The enteropathogenic *E. coli* effector EspB facilitates microvillus effacing and antiphagocytosis by inhibiting myosin function. Cell Host Microbe 2, 383–392. 10.1016/j.chom.2007.09.012 18078690

[B50] Ingle D. J. Tauschek M. Edwards D. J. Hocking D. M. Pickard D. J. Azzopardi K. I.. (2016). Evolution of atypical enteropathogenic *E. coli* by repeated acquisition of LEE pathogenicity island variants. Nat. Microbiol. 1:15010. 10.1038/nmicrobiol.2015.10 27571974

[B51] Institute for Health Metrics and Evaluation (2013). The Global Burden of Disease: Generating Evidence, Guiding Policy. Seattle, WA: IHME.

[B52] Isidean S. D. Riddle M. S. Savarino S. J. Porter C. K. (2011). A systematic review of ETEC epidemiology focusing on colonization factor and toxin expression. Vaccine 29, 6167–6178. 10.1016/j.vaccine.2011.06.084 21723899

[B53] Kaper J. B. Nataro J. P. Mobley H. L. (2004). Pathogenic *Escherichia coli*. Nat. Rev. Microbiol. 2, 123–140. 10.1038/nrmicro818 15040260

[B54] Karmali M. A. Gannon V. Sargeant J. M. (2010). Verocytotoxin-producing *Escherichia coli* (VTEC). Vet. Microbiol. 140, 360–370. 10.1016/j.vetmic.2009.04.011 19410388

[B55] Karmali M. A. Mascarenhas M. Shen S. Ziebell K. Johnson S. Reid-Smith R.. (2003). Association of genomic O island 122 of *Escherichia coli* EDL 933 with verocytotoxin-producing *Escherichia coli* seropathotypes that are linked to epidemic and/or serious disease. J. Clin. Microbiol. 41, 4930–4940. 10.1128/JCM.41.11.4930-4940.2003 14605120 PMC262514

[B56] Kimmitt P. T. Harwood C. R. Barer M. R. (2000). Toxin gene expression by shiga toxin-producing *Escherichia coli*: the role of antibiotics and the bacterial SOS response. Emerging Infect. Dis. 6, 458–465. 10.3201/eid0605.000503 10998375 PMC2627954

[B57] Konadu E. Donohue-Rolfe A. Calderwood S. B. Pozsgay V. Shiloach J. Robbins J. B.. (1999). Syntheses and immunologic properties of *Escherichia coli* O157 O-specific polysaccharide and Shiga toxin 1 B subunit conjugates in mice. Infect. Immun. 67, 6191–6193. 10531288 10.1128/iai.67.11.6191-6193.1999 PMC97014

[B58] Konadu E. Robbins J. B. Shiloach J. Bryla D. A. Szu S. C. (1994). Preparation, characterization, and immunological properties in mice of *Escherichia coli* O157 O-specific polysaccharide-protein conjugate vaccines. Infect. Immun. 62, 5048–5054. 7927787 10.1128/iai.62.11.5048-5054.1994 PMC303225

[B59] Konadu E. Y. Parke J. C. Jr. Tran H. T. Bryla D. A. Robbins J. B. Szu S. C. (1998). Investigational vaccine for *Escherichia coli* O157: phase 1 study of O157 O-specific polysaccharide-Pseudomonas aeruginosa recombinant exoprotein A conjugates in adults. J. Infect. Dis. 177, 383–387. 10.1086/514203 9466525

[B60] Kotloff K. L. Nataro J. P. Blackwelder W. C. Nasrin D. Farag T. H. Panchalingam S.. (2013). Burden and aetiology of diarrhoeal disease in infants and young children in developing countries (the Global Enteric Multicenter Study, GEMS): a prospective, case-control study. Lancet 382, 209–222. 10.1016/S0140-6736(13)60844-2 23680352

[B61] Kovacs S. D. Mullholland K. Bosch J. Campbell H. Forouzanfar M. H. Khalil I.. (2015). Deconstructing the differences: a comparison of GBD 2010 and CHERG's approach to estimating the mortality burden of diarrhea, pneumonia, and their etiologies. BMC Infect. Dis. 15:16. 10.1186/s12879-014-0728-4 25592774 PMC4305232

[B62] Larzabal M. Mercado E. C. Vilte D. A. Salazar-Gonzalez H. Cataldi A. Navarro-Garcia F. (2010). Designed coiled-coil peptides inhibit the Type III secretion system of enteropathogenic *Escherichia coli*. PLoS ONE 5:e9046. 10.1371/journal.pone.0009046 20140230 PMC2816223

[B63] Leitner D. R. Lichtenegger S. Temel P. Zingl F. G. Ratzberger D. Roier S.. (2015). A combined vaccine approach against *Vibrio cholerae* and ETEC based on outer membrane vesicles. Front. Microbiol. 6:823. 10.3389/fmicb.2015.00823 26322032 PMC4531250

[B64] Lewis S. B. Cook V. Tighe R. Schuller S. (2015). Enterohemorrhagic *Escherichia coli* colonization of human colonic epithelium *in vitro* and *ex vivo*. Infect. Immun. 83, 942–949. 10.1128/IAI.02928-14 25534942 PMC4333473

[B65] Li D. Chen Z. Cheng H. Zheng J. X. Pan W. G. Yang W. Z.. (2016). Inhibition of adhesion of enteropathogenic *Escherichia coli* to HEp-2 cells by binding of a novel peptide to EspB protein. Curr. Microbiol. 73, 361–365. 10.1007/s00284-016-1070-4 27246497

[B66] Liu J. Sun Y. Feng S. Zhu L. Guo X. Qi C. (2009). Towards an attenuated enterohemorrhagic *Escherichia coli* O157:H7 vaccine characterized by a deleted ler gene and containing apathogenic Shiga toxins. Vaccine 27, 5929–5935. 10.1016/j.vaccine.2009.07.097 19682616

[B67] Liu M. Ruan X. Zhang C. Lawson S. R. Knudsen D. E. Nataro J. P.. (2011). Heat-labile- and heat-stable-toxoid fusions (LTRG-STaPF) of human enterotoxigenic *Escherichia coli* elicit neutralizing antitoxin antibodies. Infect. Immun. 79, 4002–4009. 10.1128/IAI.00165-11 21788385 PMC3187267

[B68] Lundgren A. Bourgeois L. Carlin N. Clements J. Gustafsson B. Hartford M.. (2014). Safety and immunogenicity of an improved oral inactivated multivalent enterotoxigenic *Escherichia coli* (ETEC) vaccine administered alone and together with dmLT adjuvant in a double-blind, randomized, placebo-controlled Phase I study. Vaccine 32, 7077–7084. 10.1016/j.vaccine.2014.10.069 25444830

[B69] MacLennan C. A. Saul A. (2014). Vaccines against poverty. Proc. Natl. Acad. Sci. U.S.A. 111, 12307–12312. 10.1073/pnas.1400473111 25136089 PMC4151718

[B70] Mayr U. B. Haller C. Haidinger W. Atrasheuskaya A. Bukin E. Lubitz W.. (2005). Bacterial ghosts as an oral vaccine: a single dose of *Escherichia coli* O157:H7 bacterial ghosts protects mice against lethal challenge. Infect. Immun. 73, 4810–4817. 10.1128/IAI.73.8.4810-4817.2005 16040994 PMC1201255

[B71] Mayr U. B. Kudela P. Atrasheuskaya A. Bukin E. Ignatyev G. Lubitz W. (2012). Rectal single dose immunization of mice with *Escherichia coli* O157:H7 bacterial ghosts induces efficient humoral and cellular immune responses and protects against the lethal heterologous challenge. Microb. Biotechnol. 5, 283–294. 10.1111/j.1751-7915.2011.00316.x 22103353 PMC3815788

[B72] McKenzie R. Bourgeois A. L. Engstrom F. Hall E. Chang H. S. Gomes J. G.. (2006). Comparative safety and immunogenicity of two attenuated enterotoxigenic *Escherichia coli* vaccine strains in healthy adults. Infect. Immun. 74, 994–1000. 10.1128/IAI.74.2.994-1000.2006 16428745 PMC1360313

[B73] McKenzie R. Bourgeois A. L. Frech S. A. Flyer D. C. Bloom A. Kazempour K.. (2007). Transcutaneous immunization with the heat-labile toxin (LT) of enterotoxigenic *Escherichia coli* (ETEC): protective efficacy in a double-blind, placebo-controlled challenge study. Vaccine 25, 3684–3691. 10.1016/j.vaccine.2007.01.043 17313998

[B74] McKenzie R. Darsley M. Thomas N. Randall R. Carpenter C. Forbes E.. (2008). A double-blind, placebo-controlled trial to evaluate the efficacy of PTL-003, an attenuated enterotoxigenic *Escherichia coli* (ETEC) vaccine strain, in protecting against challenge with virulent ETEC. Vaccine 26, 4731–4739. 10.1016/j.vaccine.2008.06.064 18602960

[B75] McWilliams B. D. Torres A. G. (2014). Enterohemorrhagic *Escherichia coli* Adhesins. Microbiol. Spectr. 2, 1–19. 10.1128/microbiolspec.EHEC-0003-2013 26103974

[B76] Mejias M. P. Hiriart Y. Lauche C. Fernandez-Brando R. J. Pardo R. Bruballa A.. (2016). Development of camelid single chain antibodies against Shiga toxin type 2 (Stx2) with therapeutic potential against Hemolytic Uremic Syndrome (HUS). Sci. Rep. 6:24913. 10.1038/srep24913 27118524 PMC4847011

[B77] Messens W. Bolton D. Frankel G. Liebana E. Mc L. J. Morabito S.. (2015). Defining pathogenic verocytotoxin-producing *Escherichia coli* (VTEC) from cases of human infection in the European Union, 2007-2010. Epidemiol. Infect. 143, 1652–1661. 10.1017/S095026881400137X 25921781 PMC9507218

[B78] Monteiro R. Ageorges V. Rojas-Lopez M. Schmidt H. Weiss A. Bertin Y.. (2016). A secretome view of colonisation factors in Shiga toxin-encoding *Escherichia coli* (STEC): from enterohaemorrhagic *E. coli* (EHEC) to related enteropathotypes. FEMS Microbiol. Lett. 363:fnw179. 10.1093/femsle/fnw179 27465489

[B79] Montero D. Orellana P. Gutierrez D. Araya D. Salazar J. C. Prado V.. (2014). Immunoproteomic analysis to identify Shiga toxin-producing *Escherichia coli* outer membrane proteins expressed during human infection. Infect. Immun. 82, 4767–4777. 10.1128/IAI.02030-14 25156722 PMC4249345

[B80] Mora M. Veggi D. Santini L. Pizza M. Rappuoli R. (2003). Reverse vaccinology. Drug Discov. Today 8, 459–464. 10.1016/S1359-6446(03)02689-8 12801798

[B81] Moriel D. G. Bertoldi I. Spagnuolo A. Marchi S. Rosini R. Nesta B.. (2010). Identification of protective and broadly conserved vaccine antigens from the genome of extraintestinal pathogenic *Escherichia coli*. Proc. Natl. Acad. Sci. U.S.A. 107, 9072–9077. 10.1073/pnas.0915077107 20439758 PMC2889118

[B82] Moriel D. G. Tan L. Goh K. G. Phan M. D. Ipe D. S. Lo A. W.. (2016). A novel protective vaccine antigen from the core *Escherichia coli* genome. mSphere 1:e00326–16. 10.1128/mSphere.00326-16 27904885 PMC5120174

[B83] Nataro J. P. Kaper J. B. (1998). Diarrheagenic *Escherichia coli*. Clin. Microbiol. Rev. 11, 142–201. 9457432 10.1128/cmr.11.1.142 PMC121379

[B84] Nesta B. Spraggon G. Alteri C. Moriel D. G. Rosini R. Veggi D.. (2012). FdeC, a novel broadly conserved *Escherichia coli* adhesin eliciting protection against urinary tract infections. MBio 3:e00010-12. 10.1128/mBio.00010-12 22496310 PMC3324786

[B85] Nesta B. Valeri M. Spagnuolo A. Rosini R. Mora M. Donato P.. (2014). SslE elicits functional antibodies that impair *in vitro* mucinase activity and *in vivo* colonization by both intestinal and extraintestinal *Escherichia coli* strains. PLoS Pathog. 10:e1004124. 10.1371/journal.ppat.1004124 24809621 PMC4014459

[B86] Noguera-Obenza M. Ochoa T. J. Gomez H. F. Guerrero M. L. Herrera-Insua I. Morrow A. L.. (2003). Human milk secretory antibodies against attaching and effacing *Escherichia coli* antigens. Emerg. Infect. Dis. 9, 545–551. 10.3201/eid0905.020441 12737737 PMC2972756

[B87] Norton E. B. Lawson L. B. Freytag L. C. Clements J. D. (2011). Characterization of a mutant *Escherichia coli* heat-labile toxin, LT(R192G/L211A), as a safe and effective oral adjuvant. Clin. Vaccine Immunol. 18, 546–551. 10.1128/CVI.00538-10 21288994 PMC3122563

[B88] Ochoa T. J. Barletta F. Contreras C. Mercado E. (2008). New insights into the epidemiology of enteropathogenic *Escherichia coli* infection. Trans. R. Soc. Trop. Med. Hyg. 102, 852–856. 10.1016/j.trstmh.2008.03.017 18455741 PMC2575077

[B89] Ochoa T. J. Contreras C. A. (2011). Enteropathogenic *Escherichia coli* infection in children. Curr. Opin. Infect. Dis. 24, 478–483. 10.1097/QCO.0b013e32834a8b8b 21857511 PMC3277943

[B90] Oliveira A. F. Cardoso S. A. Almeida F. B. de Oliveira L. L. Pitondo-Silva A. Soares S. G.. (2012). Oral immunization with attenuated *Salmonella* vaccine expressing *Escherichia coli* O157:H7 intimin gamma triggers both systemic and mucosal humoral immunity in mice. Microbiol. Immunol. 56, 513–522. 10.1111/j.1348-0421.2012.00477.x 22671942

[B91] Pacheco A. R. Sperandio V. (2012). Shiga toxin in enterohemorrhagic *Escherichia coli*: regulation and novel anti-virulence strategies. Front. Cell. Infect. Microbiol. 2:81. 10.3389/fcimb.2012.00081 22919672 PMC3417539

[B92] Parissi-Crivelli A. Parissi-Crivelli J. M. Giron J. A. (2000). Recognition of enteropathogenic *Escherichia coli* virulence determinants by human colostrum and serum antibodies. J. Clin. Microbiol. 38, 2696–2700. 10878066 10.1128/jcm.38.7.2696-2700.2000 PMC87001

[B93] Pianciola L. D'Astek B. A. Mazzeo M. Chinen I. Masana M. Rivas M. (2016). Genetic features of human and bovine *Escherichia coli* O157:H7 strains isolated in Argentina. Int. J. Med. Microbiol. 32, 123–130. 10.1016/j.ijmm.2016.02.005 26935026

[B94] Pires S. M. Fischer-Walker C. L. Lanata C. F. Devleesschauwer B. Hall A. J. Kirk M. D.. (2015). Aetiology-specific estimates of the global and regional incidence and mortality of diarrhoeal diseases commonly transmitted through food. PLoS ONE 10:e0142927. 10.1371/journal.pone.0142927 26632843 PMC4668836

[B95] Pizza M. Scarlato V. Masignani V. Giuliani M. M. Arico B. Comanducci M.. (2000). Identification of vaccine candidates against serogroup B meningococcus by whole-genome sequencing. Science 287, 1816–1820. 10.1126/science.287.5459.1816 10710308

[B96] Plotkin S. (2014). History of vaccination. Proc. Natl. Acad. Sci. U.S.A. 111, 12283–12287. 10.1073/pnas.1400472111 25136134 PMC4151719

[B97] Porter C. K. Riddle M. S. Alcala A. N. Sack D. A. Harro C. Chakraborty S.. (2016). An evidenced-based scale of disease severity following human challenge with enteroxigenic *Escherichia coli*. PLoS ONE 11:e0149358. 10.1371/journal.pone.0149358 26938983 PMC4777366

[B98] Quintana Flores V. M. Campos de Souza Fernandes R. C. Sousa de Macedo Z. Medina-Acosta E. (2002). Expression and purification of the recombinant enteropathogenic *Escherichia coli* vaccine candidates BfpA and EspB. Protein Expr. Purif. 25, 16–22. 10.1006/prep.2001.1604 12071694

[B99] Rabinovitz B. C. Larzábal M. Vilte D. A. Cataldi A. Mercado E. C. (2016). The intranasal vaccination of pregnant dams with Intimin and EspB confers protection in neonatal mice from *Escherichia coli* (EHEC) O157:H7 infection. Vaccine 34, 2793–2797. 10.1016/j.vaccine.2016.04.056 27129423

[B100] Riquelme-Neira R. Rivera A. Sáez D. Fernández P. Osorio G. Del Canto F.. (2015). Vaccination with DNA encoding truncated enterohemorrhagic *Escherichia coli* (EHEC) factor for adherence-1 Gene (efa-1') confers protective immunity to mice infected with *E. coli* O157:H7. Front. Cell. Infect. Microbiol. 5:104. 10.3389/fcimb.2015.00104 26835434 PMC4718977

[B101] Rivas M. Chinen I. Guth B. E. C. (2016). Enterohemorrhagic (Shiga Toxin-Producing) *Escherichia coli*, in Escherichia coli in the Americas, ed Torres A. G. (Cham: Springer International Publishing), 97–123.

[B102] Roy K. Bartels S. Qadri F. Fleckenstein J. M. (2010). Enterotoxigenic *Escherichia coli* elicits immune responses to multiple surface proteins. Infect. Immun. 78, 3027–3035. 10.1128/IAI.00264-10 20457787 PMC2897383

[B103] Roy K. Hamilton D. J. Munson G. P. Fleckenstein J. M. (2011). Outer membrane vesicles induce immune responses to virulence proteins and protect against colonization by enterotoxigenic *Escherichia coli*. Clin. Vaccine Immunol. 18, 1803–1808. 10.1128/CVI.05217-11 21900530 PMC3209013

[B104] Roy K. Hamilton D. Ostmann M. M. Fleckenstein J. M. (2009). Vaccination with EtpA glycoprotein or flagellin protects against colonization with enterotoxigenic *Escherichia coli* in a murine model. Vaccine 27, 4601–4608. 10.1016/j.vaccine.2009.05.076 19523914

[B105] Scaletsky I. C. A. Fagundes-Neto U. (2016). Typical Enteropathogenic *Escherichia coli*, in Escherichia coli in the Americas, ed Torres A. G. (Cham: Springer International Publishing), 59–76.

[B106] Sears K. T. Tennant S. M. Reymann M. K. Simon R. Konstantopoulos N. Blackwelder W. C.. (2017). Bioactive immune components of anti-diarrheagenic enterotoxigenic *Escherichia coli* hyperimmune bovine colostrum products. Clin. Vaccine Immunol. 24:e00186-16. 10.1128/CVI.00186-16 28637804 PMC5583472

[B107] Sjöling A. von Mentzer A. Svennerholm A. M. (2015). Implications of enterotoxigenic *Escherichia coli* genomics for vaccine development. Expert Rev. Vaccines 14, 551–560. 10.1586/14760584.2015.996553 25540974

[B108] Tacket C. O. Sztein M. B. Losonsky G. Abe A. Finlay B. B. McNamara B. P.. (2000). Role of EspB in experimental human enteropathogenic *Escherichia coli* infection. Infect. Immun. 68, 3689–3695. 10.1128/IAI.68.6.3689-3695.2000 10816529 PMC97660

[B109] Tapia D. Ross B. N. Kalita A. Kalita M. Hatcher C. L. Muruato L. A.. (2016). From *in silico* protein epitope density prediction to testing *Escherichia coli* O157:H7 vaccine candidates in a murine model of colonization. Front. Cell. Infect. Microbiol. 6:94. 10.3389/fcimb.2016.00094 27625996 PMC5003871

[B110] Tarr P. I. Gordon C. A. Chandler W. L. (2005). Shiga-toxin-producing *Escherichia coli* and haemolytic uraemic syndrome. Lancet 365, 1073–1086. 10.1016/S0140-6736(05)71144-2 15781103

[B111] Taylor K. A. O'Connell C. B. Luther P. W. Donnenberg M. S. (1998). The EspB protein of enteropathogenic *Escherichia coli* is targeted to the cytoplasm of infected HeLa cells. Infect. Immun. 66, 5501–5507. 9784563 10.1128/iai.66.11.5501-5507.1998 PMC108689

[B112] Tobias J. Holmgren J. Hellman M. Nygren E. Lebens M. Svennerholm A. M. (2010). Over-expression of major colonization factors of enterotoxigenic *Escherichia coli*, alone or together, on non-toxigenic *E. coli* bacteria. Vaccine 28, 6977–6984. 10.1016/j.vaccine.2010.08.047 20728524

[B113] Tobias J. Lebens M. Bolin I. Wiklund G. Svennerholm A. M. (2008). Construction of non-toxic *Escherichia coli* and *Vibrio cholerae* strains expressing high and immunogenic levels of enterotoxigenic *E. coli* colonization factor I fimbriae. Vaccine 26, 743–752. 10.1016/j.vaccine.2007.12.009 18191006

[B114] Tobias J. Svennerholm A. M. Carlin N. I. Lebens M. Holmgren J. (2011). Construction of a non-toxigenic *Escherichia coli* oral vaccine strain expressing large amounts of CS6 and inducing strong intestinal and serum anti-CS6 antibody responses in mice. Vaccine 29, 8863–8869. 10.1016/j.vaccine.2011.09.096 21983363

[B115] Torres A. G. (2017a). *Escherichia coli* diseases in Latin America-a 'One Health' multidisciplinary approach. Pathog. Dis. 75:ftx012. 10.1093/femspd/ftx012 28158404

[B116] Torres A. G. (2017b). Maternal immunity, a wayto confer protection against enteropathogenic *Escherichia coli*. J. Pediatr. 93, 548–550. 10.1016/j.jped.2017.05.002 28602687

[B117] Turner A. K. Stephens J. C. Beavis J. C. Greenwood J. Gewert C. Randall R.. (2011). Generation and characterization of a live attenuated enterotoxigenic *Escherichia coli* combination vaccine expressing six colonization factors and heat-labile toxin subunit B. Clin. Vaccine Immunol. 18, 2128–2135. 10.1128/CVI.05345-11 21994355 PMC3232708

[B118] Turner A. K. Terry T. D. Sack D. A. Londoño-Arcila P. Darsley M. J. (2001). Construction and characterization of genetically defined aro omp mutants of enterotoxigenic *Escherichia coli* and preliminary studies of safety and immunogenicity in humans. Infect. Immun. 69, 4969–4979. 10.1128/IAI.69.8.4969-4979.2001 11447175 PMC98589

[B119] Vidal R. M. Chamorro N. L. Girón J. A. (2016). Enterotoxigenic *Escherichia coli*, in Escherichia coli in the Americas, ed Torres A. G. (Cham: Springer International Publishing), 1–26.

[B120] Wan C. S. Zhou Y. Yu Y. Peng L. J. Zhao W. Zheng X. L. (2011). B-cell epitope KT-12 of enterohemorrhagic *Escherichia coli* O157:H7: a novel peptide vaccine candidate. Microbiol. Immunol. 55, 247–253. 10.1111/j.1348-0421.2011.00316.x 21272063

[B121] Wen S. X. Teel L. D. Judge N. A. O'Brien A. D. (2006). A plant-based oral vaccine to protect against systemic intoxication by Shiga toxin type 2. Proc. Natl. Acad. Sci. U.S.A. 103, 7082–7087. 10.1073/pnas.0510843103 16641102 PMC1459021

[B122] Whitfield C. (2006). Biosynthesis and assembly of capsular polysaccharides in *Escherichia coli*. Annu. Rev. Biochem. 75, 39–68. 10.1146/annurev.biochem.75.103004.142545 16756484

[B123] Zhang C. Iqbal J. Gomez-Duarte O. G. (2016). Murine immunization with CS21 pili or LngA major subunit of enterotoxigenic *Escherichia coli* (ETEC) elicits systemic and mucosal immune responses and inhibits ETEC gut colonization. Vet Microbiol. 202, 90–100. 10.1016/j.vetmic.2016.02.001 26878971 PMC4976044

[B124] Zhang X. H. He K. W. Zhang S. X. Lu W. C. Zhao P. D. Luan X. T.. (2011). Subcutaneous and intranasal immunization with Stx2B-Tir-Stx1B-Zot reduces colonization and shedding of *Escherichia coli* O157:H7 in mice. Vaccine 29, 3923–3929. 10.1016/j.vaccine.2011.02.007 21338683

