# Correction: Intestinal pathogenic *Escherichia coli*: insights for vaccine development

**DOI:** 10.3389/fmicb.2025.1679236

**Published:** 2025-08-19

**Authors:** Maricarmen Rojas-Lopez, Ricardo Monteiro, Mariagrazia Pizza, Mickaël Desvaux, Roberto Rosini

**Affiliations:** ^1^GSK, Siena, Italy; ^2^Institut National de la Recherche Agronomique, Université Clermont Auvergne, UMR454 MEDiS, Clermont-Ferrand, France

**Keywords:** vaccines, intestinal pathogenic *E. coli* (InPEC), Enterohemorrhagic *E. coli* (EHEC), Enterotoxigenic *E. coli*, Enteropathogenic *E. coli*

Author Ricardo Monteiro was erroneously spelled as Ricardo Monterio.

The original version of this article has been updated.

